# Phosphorylation by Dyrk1A of Clathrin Coated Vesicle-Associated Proteins: Identification of the Substrate Proteins and the Effects of Phosphorylation

**DOI:** 10.1371/journal.pone.0034845

**Published:** 2012-04-13

**Authors:** Noriko Murakami, David C. Bolton, Elizabeth Kida, Wen Xie, Yu-Wen Hwang

**Affiliations:** 1 Laboratory of Molecular Regulation, New York State Institute for Basic Research in Developmental Disabilities, Staten Island, New York, United States of America; 2 Laboratory of Molecular Structure and Function/Mass Spectrometry Facility, Department of Molecular Biology, New York State Institute for Basic Research in Developmental Disabilities, Staten Island, New York, United States of America; 3 Pediatric Neuropathology, Department of Developmental Neurobiology, New York State Institute for Basic Research in Developmental Disabilities, Staten Island, New York, United States of America; Purdue University, United States of America

## Abstract

Dyrk1A phosphorylated multiple proteins in the clathrin-coated vesicle (CCV) preparations obtained from rat brains. Mass spectrometric analysis identified MAP1A, MAP2, AP180, and α- and β-adaptins as the phosphorylated proteins in the CCVs. Each protein was subsequently confirmed by [^32^P]-labeling and immunological methods. The Dyrk1A-mediated phosphorylation released the majority of MAP1A and MAP2 and enhanced the release of AP180 and adaptin subunits from the CCVs. Furthermore, Dyrk1A displaced adaptor proteins physically from CCVs in a kinase-concentration dependent manner. The clathrin heavy chain release rate, in contrast, was not affected by Dyrk1A. Surprisingly, the Dyrk1A-mediated phosphorylation of α- and β-adaptins led to dissociation of the AP2 complex, and released only β-adaptin from the CCVs. AP180 was phosphorylated by Dyrk1A also in the membrane-free fractions, but α- and β-adaptins were not. Dyrk1A was detected in the isolated CCVs and was co-localized with clathrin in neurons from mouse brain sections and from primary cultured rat hippocampus. Previously, we proposed that Dyrk1A inhibits the onset of clathrin-mediated endocytosis in neurons by phosphorylating dynamin 1, amphiphysin 1, and synaptojanin 1. Current results suggest that besides the inhibition, Dyrk1A promotes the uncoating process of endocytosed CCVs.

## Introduction

Dual-specificity tyrosine phosphorylation-regulated kinase (Dyrk1A) is a proline-directed serine/threonine kinase [1], [2], and is a highly conserved protein [3], [4], [5]. The Dyrk1A gene ortholog in Drosophila, *minibrain*, is required for the proliferation of neuroblasts during post-embryonic neurogenesis. Knock-down mutations of the gene cause a reduction in brain size of adult flies (hence, the name “minibrain"), but do not affect gross anatomy [6]. Dyrk1A is also involved in the early development of the central nervous system of vertebrates (see a review by Tejedor and Hammerle) [7]; it is expressed strongly in the central nervous system and heart, suggesting significant roles for Dyrk1A in the development of these organs. Despite its role in early development, Dyrk1A is expressed persistently throughout adulthood in the brain [8], [9], [10]. The temporal and spatial distributions of Dyrk1A suggest that this gene plays an important role in controlling adult brain functions.

Many endogenous substrates for this kinase have been identified (see a review by Aranda et al.) [11]. We have found that Dyrk1A phosphorylates multiple proteins engaged in regulated endocytosis in neurons: dynamin 1 [12], amphiphysin 1 [13], and synaptojanin 1 [14]. Dynamin 1 phosphorylation takes place primarily at Ser^857^ in the proline-rich domain (PRD) [15], which contributes to the reduction in dynamin binding to the Src homology 3 (SH3) domains of amphiphysin 1 and endophilin 1 [15]. Amphiphysin 1 is phosphorylated at the Ser^293^ within a PRD located in the middle part of the protein, and the phosphorylated amphiphysin exhibited reduced binding to endophilin 1 [13]. Phosphorylation of synaptojanin 1 by Dyrk1A also alters its binding to SH3 domains [14], yet the phosphorylation sites remain to be mapped. After exocytosis of neurotransmitters, rapid endocytosis occurs via clathrin-mediated endocytosis to recycle synaptic vesicle constituents. Our results strongly suggest that Dyrk1A functions to regulate the onset of clathrin-mediated endocytosis in neurons.

Various types of adaptor proteins and accessory proteins link clathrin to membrane cargos and lipids, and regulate clathrin coat assembly and disassembly [16], [17]. At the nerve terminus, clathrin-mediated endocytosis is regulated by many proteins, which undergo dephosphorylation-phosphorylation cycles during the exocytosis-endocytosis cycles of synaptic vesicles [18], [19]. Recently, we found that Dyrk1A was able to bind clathrin heavy chain (CHC), endophilin 1, and dynamin 1 and that Dyrk1A phosphorylated multiple proteins associated with the clathrin coated vesicles (CCVs) isolated from rat brains [20]. The current study was carried out to identify the Dyrk1A substrates associated with the brain CCVs. We report here that the substrate proteins of Dyrk1A are MAP1A, MAP2, AP180, and α- and β-adaptins and that the phosphorylation promotes dissociation of these proteins from the CCV membranes. To our knowledge, this is the first report to describe phosphorylation-dependent dissociation of clathrin-adaptor proteins from CCV membranes. Our findings suggest that Dyrk1A may regulate multiple steps of the CCV cycling processes at the onset of CCV formation and uncoating of the endocytosed CCVs.

## Materials and Methods

### Materials–(Antibodies)

Mouse monoclonal antibodies against α- and β-adaptins, SNAP91 (AP180), and MAP1A (all used for immunoprecipitation) were purchased from GeneTex (San Antonio, TX). Additional monoclonal antibodies against α- and β-adaptins, AP180, and CHC (solely used for immunoblotting) were obtained from BD Biosciences (Lexington, KY). Rabbit monoclonal anti-β-adaptin antibody was obtained from Santa Cruz (Santa Cruz, CA) whereas polyclonal and monoclonal antibodies specific to the μ subunit of the AP2 complex were from GenWay (San Diego, CA). Monoclonal anti-MAP2 antibody, rabbit polyclonal anti-N-terminal Dyrk1A (made against synthetic peptide corresponding to amino acids 32–51 of human DYRK1A), and alkaline phosphatase–conjugated goat antibodies against mouse IgG (absorbed with rat serum) as well as rabbit IgG were purchased from Sigma-Aldrich (St. Louis, MS). Monoclonal anti-Dyrk1A antibodies (8D9 and 7F3) were produced “in-house" [10], [20]. Donkey anti-mouse and anti-rabbit IgGs, conjugated with Alexa Fluor 488 and Alexa Fluor 555, respectively, and goat anti-mouse IgG conjugated with Alexa Fluor 488 were obtained from Invitrogen (Carlsbad, CA). Antibody 7F3 was directly conjugated with Alexa Fluor 568 by using a Protein Labeling Kit (Invitrogen) as suggested by the manufacturer. (*Other Reagents*): Phosphatase inhibitors (cocktail I) was purchased from Sigma-Aldrich. A complete protease inhibitor cocktail was purchased from Roche Diagnostics (Indianapolis, IN), and [γ-^32^P]-ATP was from MP Biomedicals (Costa Mesa, CA). Freshly excised rat brains were purchased from Pel-Freez (Rogers, AR) (shipped overnight on ice).

### Proteins

Full length- and truncated Dyrk1A without C-terminal PEST domain fused with GST (GST-Dyrk1A^497^) were generated and prepared as described [12], [20], [21]. Dyrk1A^497^, harboring Y319F and Y321F mutations, was prepared as previously described [22]. CCVs and microtubules (MTs) were prepared from the rat brains according to the methods described by Campbell et al. [23] and Pedrotti & Islam [24], respectively, and stored at −80°C until use. Protein concentrations were measured by the Lowry method using BSA as a standard.

Tris-HCl extract and cytosol were prepared as follows: rat brain was homogenized in five volumes of MES buffer (0.1 M MES at pH 6.5, 0.5 mM MgCl_2_, and 1 mM EGTA) [23] containing a cocktail of protease inhibitors and centrifuged first at 1000× g for 10 min. The supernatant (S1) was then centrifuged at 100,000× g for 45 min, and both precipitated (post-nuclear precipitates, *PNPs*) and supernatant fractions were collected. PNPs were suspended in a small volume of 10 mM Tris-HCl (pH 7.4) containing the protease inhibitors. The supernatant fraction was mixed with ammonium sulfate at a 66% saturation at 4°C, and the resultant aggregates were collected by centrifugation at 20,000× g for 30 min, suspended in a small volume of dialysis buffer (20 mM Tris-HCl at pH 7.4, 0.5 mM DTT, 0.5 mM EDTA, 0.5 mM EGTA, and 0.12 M NaCl), and dialyzed overnight against the same buffer. The soluble fraction (*cytosol*) was obtained by microcentrifuging the dialysate at maximum speed for 20 min. The suspended PNPs were mixed with 0.5 M Tris-HCl (pH 7.4), 1 mM EDTA, and protease inhibitors at 4°C for 1 hr to strip the membrane-bound proteins. After centrifugation at 100,000× g for 45 min, the supernatant fraction (*PNP extract*) was collected. Both cytosol and PNP extract were stored at −80°C. Immediately before use, the PNP extract was diluted with 5 mM MgCl_2_, 0.12 M NaCl, 0.1 mM EGTA, 0.2 mM DTT, and a cocktail of protease inhibitors to reduce the Tris-HCl concentration to 0.125 M. Both cytosol and the diluted PNP extract were clarified by ultracentrifugation at 100,000× g before use to remove precipitates. Where indicated, an aliquot of S1 described above was centrifuged to obtain P2 (precipitates at 19,000× g for 40 min) and P3 (precipitates at 100,000× g for 1 hr of the 19,000× g supernatant). These precipitates were re-suspended in the homogenization buffer.

### Phosphorylation Assay

The standard reaction was carried out at 30°C for 30 min by incubating 3–20 µg proteins with 0.5–3.7 µg GST-Dyrk1A^497^ and 0.1–0.2 mM [γ-^32^P]-ATP (0.5–1 µCi/tube) or 1–2 mM cold ATP in kinase buffer (25 mM of HEPES at pH 7.4, 5 mM MgCl_2_, 0.1 M NaCl, and 0.1 mM DTT) in a final volume of 30 µl, unless otherwise indicated. Protease inhibitors were always included in the reaction mixtures. After the reaction was stopped by adding 10 mM EDTA, a mixture of phosphatase inhibitors (Na_3_VO_4_, Na_2_MoO_4_, okadaic acid, and phosphatase inhibitor cocktail I) was added. The reaction mixtures were either boiled in SDS-sample buffer or ultracentrifuged to separate the proteins dissociated from the CCV membranes, unless otherwise specified. A mixture of phosphatase and protease inhibitors was present in all SDS sample buffers for the phosphorylated samples.

### SDS-PAGE and Western Blotting

SDS-PAGE was carried out according to the method of Schagger [25] using either mini- (10 cm×7 cm) or regular-size gels (14 cm×15 cm) composed of 7% acrylamide and 0.128% *bis*-acrylamide. Proteins separated by SDS-PAGE were either stained by Coomassie Blue or transferred to PVDF membranes. The membranes were either stained directly with Coomassie Blue or blocked with 5% milk followed by immunoblotting. The membranes with the transferred [^32^P]-labeled proteins were processed for autoradiography first and then subjected to immunoblotting, or *vice versa*. In the former case, the PVDF membranes were sealed within plastic envelopes containing a small volume of 5% milk and exposed to x-ray films at −80°C; they were then subjected to immunoblotting by using the appropriate antibody. The antibody-reactive bands were visualized by either color development or chemiluminescence detection using CDP star reagent (New England BioLabs; Ipswich, MA).

### Mass Spectrometric (MS) Analysis of the Proteins Phosphorylated by Dyrk1A

CCVs in duplicate tubes were incubated with recombinant GST-Dyrk1A^497^ in the standard assay conditions with cold- or [γ-^32^P]-ATP. After SDS-PAGE using regular-size gels, the [^32^P]-labeled samples were stained with Coomassie Blue, dried, and subjected to autoradiography to locate the positions of the [^32^P]-labeled protein bands. Each band corresponding to the [^32^P]-labeled proteins was cut out from the gels containing cold-labeled samples and processed for LC-MS/MS analysis to identify the phosphorylated proteins according to the methods described previously [20].

### Immunoprecipitation

Immobilized protein A/G (30 µl gel suspension) (Thermo Scientific; Rockford, IL) was washed, suspended in a small volume of phosphate-buffered saline with 0.05% Tween-20 (PBS-T), and incubated with a primary antibody at room temperature first for 10 min, and then at 4°C for 1 h by adding 50 µl of 5% BSA in PBS-T (BSA blocking buffer). For immunoprecipitation of MAP2 and MAP1A, the soluble proteins after phosphorylation of MTs were collected by ultracentrifugation and used directly with appropriate antibodies. The CCV-associated proteins were stripped from the vesicles, either before or after phosphorylation reaction, by incubation with 0.5 M Tris-HCl (pH 7.5) and 1 mM EDTA as described above in the presence of various protease and phosphatase inhibitors. The Tris-HCl concentrations of the CCV- and PNP-extracts were diluted by adding 3 volumes of PBS-T containing various protease inhibitors (and phosphatase inhibitors where appropriate). To the CCV extract, BSA (1 mg/ml) was added before immunoprecipitation. For immunoprecipitating the AP2 complex from cytosol, Tris-HCl at 0.125 M was added prior to mixing with the protein A/G resins pre-incubated with an appropriate antibody. Following the incubation at 4°C overnight, the protein A/G resins were washed three times, by brief centrifugation and suspension in 1 ml each of IP buffer (1% Triton X-100, 0.15 M NaCl, 10 mM Tris-HCl at pH 7.4, 0.1 mM EGTA, and 0.5% NP-40) containing various protease and phosphatase inhibitors. The proteins recovered with the resins were subjected to SDS-PAGE followed by Coomassie-Blue staining, autoradiography, and immunoblotting. Alternatively, the immunoprecipitates were subjected to phosphorylation by Dyrk1A. Where indicated, the stained gels or immunoblots were scanned using an Epson V700 scanner, and the staining intensities of each band were quantified using Image-J (National Institutes of Health, Bethesda, MD).

### Cell Culture

CHO and PC 12 cells were obtained from ATCC (Manassas, VA). CHO and PC12 cells were grown in DMEM containing 10% fetal calf serum (FCS) and 12.5% horse serum plus 2.5% FCS, respectively. Four hrs after exchanging with fresh media, the cells were rinsed with cold PBS and harvested by scraping into Beckman microcentrifuge tubes. The cell pellets were suspended in small volumes (75–200 µl) of AP complex-extraction buffer (20 mM HEPES at pH 7.4, 1 mM EGTA, 0.12 M NaCl, 2.5 mM MgCl_2_, and 0.2 mM DTT) containing cocktails of various protease- and phosphatase inhibitors. The cell suspensions were frozen and thawed 10 times, and centrifuged at 100,000× g for 45 min to obtain soluble and insoluble fractions. The insoluble fractions were suspended, by sonication, into the original volumes of AP complex-extraction buffer containing a cocktail of protease inhibitors. The volumes of the suspended precipitates were adjusted to match the volumes of supernatants. E18 primary rat hippocampal neurons purchased from Genlantis (San Diego, CA) were cultured in B27/Neurobasal medium with 0.5 mM glutamine and 25 µM glutamate for 4 days and then without glutamate for another 6 days, as per provider instructions.

### Immunostaining of Brain Sections and Primary Cultured Neurons

Animals were treated under a protocol that was approved by the Institutional Animal Care and Use Committee of the New York State Institute for Basic Research in Developmental Disabilities and conforms to the guidelines set forth in the *Guide for the Care and Use of Laboratory Animals* (National Research Council). Adult female mice (B6EiC3) (Jackson Laboratory; Bar Harbor, ME) were perfused with 0.5% glutaraldehyde/2% formaldehyde in 0.1 M PBS, and various parts of the brain were dissected out as described before [26]. Immunofluorescence was performed on 40-µm free-floating Vibratome sections, according to the methods described earlier [26] but with modifications. In brief, each brain section was blocked for 4 hr at room temperature in 10% FCS/PBS with 0.1% saponin, then incubated for 24 hr at 4°C with primary antibodies diluted in 10%FCS/PBS/1% saponin. Dilution ratios for rabbit polyclonal anti-N-terminal Dyrk1A and mouse monoclonal anti-CHC antibodies were 1∶500 and 1∶200, respectively. After several rinses, sections were incubated for 4 hr with species-specific secondary antibodies (from a donkey source) conjugated with Alexa Fluor 488 (1∶1,000 dilution) and Alexa Fluor 555 (1∶2,000 dilution) for mouse and rabbit IgG, respectively. After several rinses, the sections were mounted on microscope slides and cover slipped with mounting medium (Vector Laboratories Inc.; Burlingame, CA). Differentiated hippocampal neurons as described above were washed once with PBS and fixed with 2% formaldehyde in PBS for 20 min at room temperature followed by incubation in blocking buffer (PBS with 2% BSA, 2% goat serum, and 0.05% saponin) for 1 hr. Cells were first incubated with anti-CHC antibody (1∶250 dilution) for 1 hr and then with Alexa Fluor 488-conjugated goat anti-mouse IgG (1∶500 dilution) for another 1 hr. Afterward, cells were washed 3 times with PBS/0.05% saponin, blocked again for 1 hr, and incubated with Alexa Fluor 568-conjugated anti-Dyrk1A (7F3, 3 µg/ml in blocking buffer) for 1 hr to complete the double staining. Fluorescent images were captured with a Nikon C1 laser-scanning confocal system mounted on Nikon 90i microscope. Where indicated, Z-stack major projections were generated by collecting images at 0.15-µm steps along the Z-axis. Optical images were processed by using Adobe Photoshop 6.0 and Image J software. Omission of the primary antibodies served as a control of the method specificity.

## Results

### Identification of the Dyrk1A Substrates Associated with Brain CCVs

**Figure 1:**
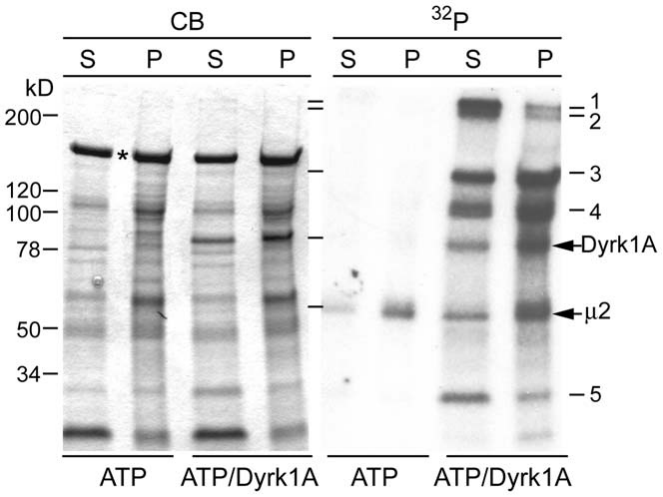
Dissociation of the phosphorylated proteins from CCVs. Rat brain CCVs (20 µg) were incubated with 0.1 mM [γ-^32^P]-ATP with or without GST-Dyrk1A^497^ (3.7 µg) as described in *MATERIALS AND METHODS*. After mixing with EDTA and phosphatase inhibitors, the samples were transferred on ice and ultracentrifuged at 70,000 rpm for 15 min using a Beckman TLA-100 rotor. The supernatants (*S*) were collected, and the precipitates (*P*) were suspended in the original volume of the kinase buffer containing protease- and phosphatase- inhibitors. Each sample was subjected to SDS-PAGE followed by Coomassie Blue staining (*CB*) and autoradiography (*^32^P*). Half of each SDS sample was applied per lane. The numbers in the right panel refer to the phosphorylated protein bands 1–5. Dyrk1A^497^ was used in most of our experiments, because this truncated form 1) is highly purified in contrast to the full-length protein, which always contains kinase bands degraded to various extents [20], and 2) exhibits the similar kinase activity as the full-length protein [21]. *, denotes clathrin heavy chain (CHC). (*n = 3; the assay was repeated three times by using different CCV preparations giving similar results*.)

Various proteins required for CCV formation are known to be phosphoproteins [27]. In an earlier study, we found that recombinant Dyrk1A phosphorylated multiple proteins in a brain CCV preparation [20]. To test whether these proteins exhibit altered binding to the vesicles upon phosphorylation by Dyrk1A, we analyzed the recovery of the [^32^P]-labeled proteins in the soluble (*S*) and precipitated (*P*) fractions (Fig. 1). In agreement with our previous results, we identified five distinctive protein bands phosphorylated by Dyrk1A (*panel^ 32^P, ATP/Dyrk1A*). Without exogenous kinase, only one band of around 58 kDa was [^32^P]-labeled (*ATP*); this protein was assumed to be μ-adaptin of the AP2 complex phosphorylated by cyclin G-associated kinase (GAK)/auxilin 2 [28], a kinase that purifies with the CCVs. After incubation with recombinant Dyrk1A and [γ-^32^P]-ATP, most of the radioactivity associated with bands 1 and 2 (estimated molecular sizes 400 kDa and 350 kDa, respectively) and band 5 (approximately 32 kDa) was recovered in the supernatant. Protein bands 3 and 4 (estimated molecular weights 145 kDa and 110 kDa, respectively), autophosphorylated Dyrk1A, and the endogenously phosphorylated μ-adaptin were partially recovered in the supernatant (*lanes S*). Obviously, substantial levels of clathrin heavy chain (CHC) (*, *panel CB*) were dissociated from CCVs under the assay conditions, regardless of the presence of exogenous Dyrk1A.

**Figure 2:**
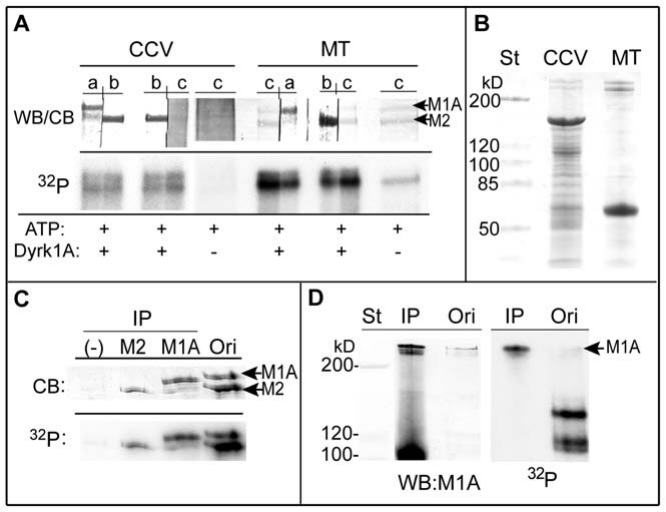
Identification of the phosphorylated protein bands 1 and 2 as MAP1A and MAP2. (***A***) *MAP1A and MAP2 in the phosphorylated CCVs and MTs*. CCVs (20 µg) and purified MTs (5 µg) were incubated with [γ-^32^P]-ATP with (+) or without (−) GST-Dyrk1A^497^ as described in Fig. 1, followed by SDS-PAGE without ultracentrifugation. Approximately 9 µg and 2 µg of CCVs and MTs, respectively, were applied per lanes. After transferring proteins, each lane of the PVDF membranes was cut into two strips for immunostaining either with anti-MAP1A (*a*) or anti-MAP2 (*b*) antibody, or for Coomassie Blue staining (*c*). The strips were reassembled (*WB/CB*) and subjected to autoradiography (*^32^P*). (*n = 2*). (***B***) *Coomassie Blue-staining of the CCV and MT preparations*. Ten and five µg of CCVs and MTs, respectively, were applied per lane. (***C***) *Immunoprecipitation of MAP1A and MAP2 from the phosphorylated MTs*. MTs (200 µg) were phosphorylated for 1 hr with GST-Dyrk1A^497^ (18 µg) and 0.2 mM [γ-^32^P]-ATP in a final volume of 250 µl. After the reaction, the soluble fraction was subjected to immunoprecipitation (*IP*) by using anti-MAP2 (*M2*) or anti-MAP1A (*M1A*) antibody as described in *MATERIALS AND METHODS*. A negative control for the immunoprecipitation (−) was obtained without primary antibody. The immunoprecipitates were applied to SDS-PAGE followed by Coomassie Blue staining (*CB*) and autoradiography (*^32^P*). (*n = 1; various preliminary performances carried out to lead the final assay conditions are not included*). Scanning of the MAP1A and MAP2 bands from the original material used for immunoprecipitation (*Ori*) gave the arbitrary units for these proteins as 3306 and 6323, respectively, whereas those for the radioactivity were 6056 and 12582, respectively. (***D***) *Immunoprecipitation of MAP1A from the extract of the phosphorylated CCVs*. CCVs (60 µg) were incubated with GST-Dyrk1A^497^ (7 µg) and 0.2 mM [γ-^32^P]-ATP for 1 hr in a final volume of 120 µl. The phosphorylated CCVs were extracted with 0.5 M Tris-HCl, diluted the Tris-HCl concentration, and used for immunoprecipitation with anti-MAP1A antibody (*IP*). The immunoprecipitates were subjected to blotting (*WB*) with anti-MAP1A antibody followed by autoradiography (*^32^P*). (*n = 2*). *St*, pre-stained standard proteins; *M1A*, MAP1A; *M2*, MAP2.

We then aimed to identify the Dyrk1A substrates in the CCV preparations by using the supernatant fractions from the phosphorylated CCVs because this fraction had cleaner background staining. Phosphorylation reactions were carried out in parallel by using [γ-^32^P]-ATP and cold ATP. After autoradiography, the protein bands corresponding to each radioactive band were located in the gels of the cold-labeled samples to excise for MS analysis. The results are summarized in Table 1. MS analyses of multiple sets of the samples identified the phosphorylated protein in band 1 as MAP1A. CHC was ruled out from band 1 because of its molecular weight. Besides, its intrinsic band was not phosphorylated by Dyrk1A (*, Fig. 1). Similarly, MS analysis revealed that band 2 consisted of both MAP2 and MAP1A. MAP1A contains the consensus sequences for Dyrk1A phosphorylation (RPXSP and RXSP) [1] at multiple locations, whereas MAP2 does not. Accordingly, we tested whether MAP1A and MAP2 were indeed substrates for Dyrk1A, by [^32^P]-phosphorylating both CCVs and purified microtubules (MTs) from rat brains and subjecting them to Western blotting followed by autoradiography (Fig. 2*A*). After the proteins were transferred to PVDF membranes, single lanes were cut into two strips; the strips were probed with either an antibody against MAP1A (*a*) or MAP2 (*b*), or stained with Coomassie Blue (*c*) (*panel WB/CB*). Immunoblotting revealed that the CCV preparations contained both MAP1A and MAP2, and autoradiography suggested that both protein bands were [^32^P]-labeled only when Dyrk1A was added (*panel ^32^P*). Immunoblotting also showed that there was some degraded MAP1A located in close proximity to the MAP2 band (*CCV*, *strip a*). As seen in Fig. 2*B*, after Coomassie Blue staining of a polyacrylamide gel, two bands of high molecular-weight proteins were clearly identified for both the CCVs and MTs at positions where MAP1A and MAP2 would be expected to migrate. In addition, the discrete bands of MAP1A and MAP2 from MTs visualized by Coomassie-Blue staining of the PVDF membranes were matched to the bands revealed by immunostaining (*strips a-c*, *panel WB/CB, MT*) (Fig. 2*A*). The MAP2 band from MTs had detectable basal phosphorylation by endogenous kinase(s) (*panel ^32^P*). Addition of Dyrk1A significantly enhanced the [^32^P]-phosphate incorporation into the bands matched with the position of MAP1A and MAP2.

**Table 1:** Summary of the MS analysis for identification of the rat proteins in the Coomassie Blue-stained bands.

Band # (size)	Accession Number*	Protein identified	(Peptides)	% Seq.
*Sample 1*				
Band 1 (400 kDa):	NP_112257.1	MAP1A	(4)	1.7
	NP_062172.1	Clathrin heavy chain	(3)	2.1
Band 2 (350 kDa):	*No data*			
Band 3 (145 kDa):	NP_113916.1	SNAP 91/AP180	(5)	4.7
Band 4 (110 kDa):	NP_001100981.1	AP2 α1-adaptin	(6)	6.6
	NP_058973.1	AP1 β1-adaptin	(5)	6.6
	NP_542150.1	AP2 β1-adaptin	(4)	5.2
	NP_062172.1	Clathrin heavy chain	(2)	1.4
	NP_001101419.1	DNAJ Hsp40 homolog	(2)	2.4
Band 5 (30 kDa):	*No data*			
*Sample 2*				
Band 1 (400 kDa):	NP_112257.1	MAP1A	(13)	5.5
	NP_062172.1	Clathrin heavy chain	(8)	4.5
Band 2 (350 kDa):	NP_062172.1	Clathrin heavy chain	(3)	1.8
	NP_112257.1	MAP1A	(3)	1.4
	NP_037198.1	MAP2	(2)	1.4
Band 3 (145 kDa):	NP_062172.1	Clathrin heavy chain	(24)	12.0
	NP_113916.1	SNAP 91/AP180	(9)	7.4
	NP_476459.1	Contactin 1 precursor	(5)	4.4
Band 4 (110 kDa):	NP_112270.2	AP2 α2-adaptin	(9)	8.9
	NP_542150.1	AP2 β1-adaptin	(6)	7.0
	NP_001100981.1	AP2 α1-adaptin	(4)	3.2
Band 5 (30 kDa):	NP_062039.1	Dyrk1A	(5)	14.1
	GT26_SCHJA	Synaptogyrin 1	(2)	10.3
		Glutathione S transferase†	(14)	36.7
*Sample 3*				
Band 1 (400 kDa):	NP_112257.1	MAP1A	(4)	1.6
Band 2 (350 kDa):	NP_062172.1	Clathrin heavy chain	(3)	1.8
Band 3 (145 kDa):	NP_113916.1	SNAP 91/AP180	(8)	6.6
	NP_062172.1	Clathrin heavy chain	(7)	4.0
	NP_112399.1	Tripeptidyl peptidase II	(5)	4.1

* All database searches were performed using the rat-specific RefSeq database (version 9-21-2009).

† The identifications of glutathione S transferase (GST) and the GST-Dyrk1A fusion protein were made from searches of the SwissProt database (version circa 2005) and a version of that database containing several GST fusion protein sequences, including GST-Dyrk1A.

The phosphorylation of MAP1A and MAP2 by Dyrk1A was further confirmed by immunoprecipitating these proteins from the soluble fraction obtained after phosphorylating MTs (Fig. 2*C*). Coomassie Blue staining (*CB*) and autoradiography (*^32^P*) confirmed that the MAP1A and MAP2 bands containing [^32^P]-phosphate were immunoprecipitated by the antibodies specific to each protein (*M1A and M2*). The phosphorylation efficiencies of MAP1A and MAP2 in MTs were estimated semi-quantitatively by measuring the relative intensities of each band from protein staining (*CB*) and from autoradiogram (*^32^P*) of the original MT extract (*lane Ori*). The MAP1A to MAP2 ratio measured by protein staining was calculated to be 1∶1.9 whereas the ratio measured by phosphorylation was 1∶2.1. This suggests that Dyrk1A phosphorylated MAP1A and MAP2 in MTs with similar efficiencies. By confirming that both MAP1A and MAP2 are equally phosphorylatable by Dyrk1A, we next verified, by immunoprecipitation, that the phosphorylated protein bands 1 and 2 in the CCV preparations were the MAP proteins. After incubating the stripped proteins from the [^32^P]-phosphorylated CCVs with anti-MAP1A antibody, one major band that reacted with the antibody and contained the radioactivity was observed (Fig. 2*D*). Intensities of the bands from immunoblots (*WB*) and autoradiogram (*^32^P*) were significantly increased after immunoprecipitation (*IP*) from the starting materials (*Ori*). Without the primary antibody, no band was detected (not shown). Therefore, we concluded that the phosphorylated protein in band 1 from brain CCVs (Fig. 1) was MAP1A. In contrast to the result obtained from MTs (Fig. 2*C*), so far we were unable to immunoprecipitate MAP2 from the CCV extracts, even when we employed the same antibody as in Fig. 2C (made against rat brain MAPs) at higher concentrations or used a different source of the antibody (made against peptide aa1–300 of human MAP2) (data not shown). Regardless, we concluded that band 2 (Fig. 1) contained the phosphorylated MAP2 because 1) Dyrk1A phosphorylated MAP2 equally to MAP1A, 2) brain CCVs contained both MAP1A and MAP2, and 3) multiple peptides derived from MAP2 were identified by MS from band 2 (Table 1). We speculate that the sites on MAP2 recognized by these antibodies may be blocked in the CCV extract, thus preventing precipitation.

**Figure 3:**
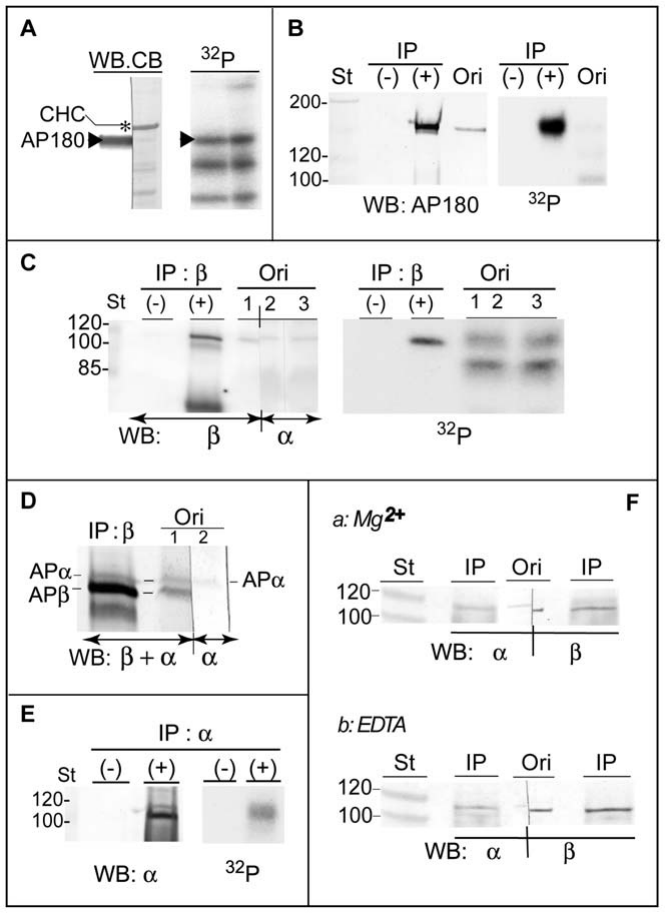
Identification of the phosphorylated protein bands 3 and 4. (***A***) *Migration patterns of AP180 and band 3 in SDS-PAGE*. After [^32^P]-phosphorylation, CCVs were subjected to SDS-PAGE followed by blotting with anti-AP180 antibody (WB), Coomassie-Blue staining (*CB*), and autoradiography (*^32^P*) as in Fig. 2*A*. (*n = 2*). Approximately 1.3 µg proteins were applied per lane, and a single lane of the PVDF membrane was cut into two strips. Arrowheads, AP180; asterisk, CHC. Two lower bands in the autoradiogram are band 4 and the autophosphorylated GST-Dyrk1A^497^. (***B***) *Immunoprecipitation of AP180 from the extract of the phosphorylated CCVs*. CCVs were incubated with GST-Dyrk1A^497^ and [γ-^32^P]-ATP, extracted with Tris-HCl, and used for immunoprecipitation (*IP*) using anti-AP180 antibody (anti-SNAP91), as described in Fig. 2*D*. The resultant immunoprecipitates were subjected to blotting using anti-AP180 antibody (*WB:AP180*) followed by autoradiography (*^32^P*). (*n = 2*). (*−*) and (*+*), immunoprecipitation without and with anti-AP180 antibody, respectively; *Ori*, the CCV extract used for immunoprecipitation. *(* ***C*** *) Immunoprecipitation of β-adaptin*. The CCV extract prepared as in *(B)* was subjected to immunoprecipitation without (−) or with (+) anti-β-adaptin antibody (*IP:β*). (*n = 2*). One lane of the starting extract (*Ori*) was cut into two strips; one was probed with anti-β-adaptin (*strip 1*) together with the immunoprecipitates (*WB:β*), and the other (*strip 2*) and lane *3* were incubated with anti-α-adaptin antibody (*α*). All strips were reassembled for autoradiography (*^32^P*). The lower band in the WB panel is IgG heavy chain. *(* ***D*** *) Immunoprecipitation of β-adaptin but not α-adaptin by anti-β-adaptin antibody*. The membranes containing *lane (+)* and *strip 1* from *(C)* were re-blotted with anti-α-adaptin (*WB:β+α*) and re-assembled with strip *2* from (*C*). The ratios in relative intensities of α- (*APα*) to β (*APβ*)-adaptins in the *IP:β* lane and *strip 1* shown in here were 1∶40.5 and 1∶2.7, respectively. *(* ***E*** *) Immunoprecipitation of α-adaptin*. After the first immunoprecipitation using anti-β-adaptin antibody *(C)*, the unbound fraction was incubated with (+) or without (−) anti-α-adaptin antibody (*IP:α*) for the second immunoprecipitation. The precipitates were subjected to immunoblotting using anti-α-adaptin antibody (*WB:α*) followed by autoradiography (*^32^P*). (*n = 1*). *(* ***F*** *) Co-precipitation of α- and β-adaptins from the extracts of unphosphorylated CCVs*. CCVs in two tubes were diluted in kinase buffer, mixed with either H_2_O (*Mg^2+^*) or 10 mM EDTA, and extracted with 0.5 M Tris-HCl for immunoprecipitation with anti-β-adaptin antibody as in *(C)*. (*n = 3*). The immunoprecipitates (*IP*) and the original extracts (*Ori*) were blotted using antibodies against α- or β-adaptin (*WB: α, β*).

The phosphorylated protein in band 3 (145 kDa) was identified by MS as SNAP91/AP180 (AP180). Like MAP2, the amino acid sequence indicates that AP180 lacks a consensus Dyrk1A phosphorylation site. Therefore, the MS result was verified, first, by subjecting the [^32^P]-labeled CCVs to SDS-PAGE followed by immunoblotting, Coomassie Blue staining, and autoradiography. A single lane cut into two pieces was stained with either anti-AP180 antibody (*WB*) or Coomassie Blue (*CB*) (Fig. 3*A*). A band that strongly reacted with anti-AP180 antibody (*arrowhead*) matched well with a protein band faintly stained by Coomassie Blue, located below the major band of CHC (*). Autoradiography of the strips (*^32^P*) indicates that the band recognized by the antibody was [^32^P]-labeled (*arrowhead*). Next, we carried out an immunoprecipitation assay using the stripped proteins from the [^32^P]-phosphorylated CCVs by using the same strategy as that used for MAP1A. Anti-AP180 antibody precipitated a single protein band containing the radioactivity (Fig. 3*B*). The intensities of the band in both immunoblots (*WB*) and the autoradiogram (*^32^P*) were significantly increased after immunoprecipitation (*+*) from the starting materials (*Ori*). Therefore, we concluded that band 3 was AP180.

The MS analyses suggest that the 110-kDa proteins in band 4 contained α1- and/or α2-adaptins from the AP2 complex and β1- and/or β2- adaptins from the AP1 and AP2 complexes. The results were verified, again, by immunoprecipitation. Anti-β-adaptin antibody, which recognizes both β1- and β2 isoforms, successfully precipitated one major band of β-adaptin (*WB:β*) containing [^32^P]-phosphate (*^32^P*) (Fig. 3*C*, *lanes +*). Without the antibody, no radioactivity was detected. To show the relative positions of α- and β-adaptins separated by SDS-PAGE, a single lane of the original CCV extract was cut into two strips, which were incubated with antibodies specific to either anti-β- (*strip 1*) or anti-α-adaptin (*strip 2*) antibody. Although α- and β-adaptins migrated in close proximity, they were clearly separated (*WB*). Autoradiography of the original extract showed a diffused band that overlapped both α- and β-adaptins (*^32^P*, *lanes 1–3*). To analyze the extent that the co-precipitated α-adaptin contributed to the radioactive band, the same blot from Fig. 3*C* [*lanes (+) & strip 1*] was re-probed with anti-α-adaptin antibody. The latter blotting detected a new band of *α*-adaptin in the original CCV extract (Fig. 3*D* *, strip 1, β+α*) at the same position recognized by anti-α-adaptin antibody (*α*) alone (*strip 2*). However, to our surprise, the re-blotting revealed that the immunoprecipitation of β-adaptin did not increase the α-adaptin level in proportion to the enhanced β-adaptin level. A ratio of α- to β-adaptin in strip 1 was roughly estimated to be 1∶2.7, whereas that in lane (*IP:β*) was at least 1∶40.5. These results suggest that dissociation between α-adaptin and β-adaptin occurred upon phosphorylation with Dyrk1A. Therefore, α-adaptin was immunoprecipitated from the resulting unbound fraction after the first immunoprecipitation for β-adaptin and tested to determine if the precipitated protein was also [^32^P]-phosphorylated. We found that anti-α-adaptin antibody precipitated two anti-α-adaptin antibody-reacting bands from the anti-β-adaptin unbound fraction (Fig. 3*E* *, WB:α*). Autoradiography gave a diffuse band covering the area of the two bands (*^32^P*). Thus, we confirmed α-adaptin also as a Dyrk1A substrate.

Under native conditions, α-, β2-, μ2-, and σ2-adaptins associate together to form the AP2 complex. Because EDTA was added to terminate the phosphorylation reaction, we tested whether EDTA caused the dissociation of AP2 complex. The proteins stripped from the unphosphorylated CCVs by 0.5 M Tris-HCl with either 2.5 mM Mg^2+^ or 5 mM EDTA were subjected to immuno-precipitation using anti-β-adaptin antibody. The resultant precipitates together with the original extracts were immunoblotted with anti-α- and β-adaptin antibodies (Figure 3*F*). Overall immunoprecipitation efficiencies of the Tris-HCl extracts from the un-phosphorylated CCVs were low; however, the anti-β-adaptin antibody precipitated not only β-adaptin but also α-adaptin, regardless of the presence of Mg^2+^ or EDTA (Fig. 3*F*). Furthermore, the levels of α-adaptin co-precipitated with β-adaptin did not appear to be different between the two samples, indicating that the apparent lack of co-precipitation of α- and β-adaptins observed in the Dyrk1A-phosphorylated CCVs (Fig. 3*D*) was not due to the EDTA-mediated dissociation of the AP2 complex. In summary, we conclude that band 4 in Fig. 1 consists of both α- and β-adaptins and that most of these adaptins are dissociated from each other upon phosphorylation by Dyrk1A. Protein band 5 was identified as a fragmented GST-Dyrk1A (Table 1).

### Recovery of MAP1A and MAP2 in the Soluble Fraction

**Figure 4:**
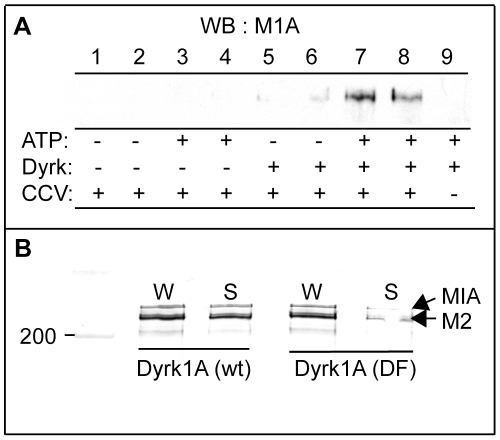
Release of MAP1A and MAP2 into the soluble fraction after phosphorylation. (***A***) *Release of MAP1A*. CCVs (20 µg) were phosphorylated and ultracentrifuged as described in Fig. 1 except for using 2 mM cold ATP and with or without GST-Dyrk1A^497^. The resultant supernatants were blotted with anti-MAP1A antibody *(M1A).* (*n = 3*). (***B***) *Phosphorylation-dependent dissociation of MAP1A and MAP2.* CCVs were incubated as in *(A)* in duplicate tubes containing ATP and either wild type *(wt)* or double mutant (*DF*) GST-Dyrk1A^497^. After adding EDTA, one set of tubes was kept on ice, whereas the other set was ultracentrifuged to collect the soluble fractions. Aliquots of whole mixtures (*W*) and the supernatants (*S*) were subjected to immunoblotting. Both MAP1A *(M1A)* and MAP2 *(M2)* were identified in the same lane by blotting the PVDF membrane first with anti-MAP2 then with anti-MAP1A antibodies. (*n = 1, various preliminary performances carried out to lead the final assay conditions are not included*).

As seen in Fig. 1, significant amounts of the [^32^P]-labeled proteins were found in the soluble fraction. Therefore, we analyzed the effect of Dyrk1A on the release of MAP1A and MAP2 from CCVs. Neither MAP1A (Fig. 4*A*, *lanes 1–4*) nor MAP2 (not shown) was recovered in the soluble fraction if exogenous Dyrk1A was omitted. Addition of Dyrk1A and ATP caused a release of MAP1A into the soluble fraction (*lanes 7 & 8*). The kinase alone also released MAP1A but at much lower levels (*lanes 5 & 6*). When MTs were used under the similar assay conditions, in contrast to CCVs, both MAP1A and MAP2 were released in the soluble fraction in an ATP-dependent but Dyrk1A-independent manner (data, not shown).

The effect of the Dyrk1A-mediated phosphorylation on dissociation of MAP proteins from CCVs was further studied by measuring the MAP2 and MAP1A levels in the whole reaction mixtures (*W*) and in the soluble fraction (*S*) after phosphorylation reaction with either wild-type (*wt*) or a kinase-defective mutant harboring Y319F and Y321F double mutation (*DF*) [22] (Fig. 4*B*). In the presence of ATP, Dyrk1A-wt released most MAP1A and MAP2 from the CCVs whereas the DF mutant released only small amounts of the MAP proteins, suggesting that the release of MAP proteins from CCVs required Dyrk1A activity and occurred mostly after phosphorylation.

### Dissociation of the Clathrin Adaptor Proteins from CCVs

**Figure 5:**
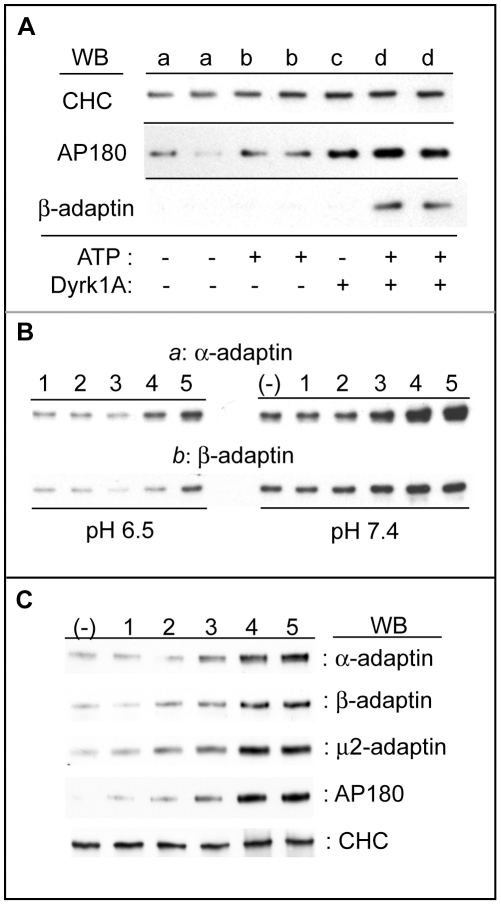
Effect of Dyrk1A on dissociation of adaptor proteins from CCVs. *(* ***A*** *) Effect of phosphorylation.* CCVs (3.5 µg) were incubated with ±1 mM cold ATP and ± Dyrk1A (0.5 µg), as indicated in the panel, and ultracentrifuged as described in Fig. 1. The resultant supernatants were immunoblotted (*WB*) using the antibodies indicated in the figure. (*n = 2*). *(* ***B*** *) Release of α- and β-adaptins by Dyrk1A without phosphorylation*. CCVs (3.75 µg) were incubated with various amounts of GST-Dyrk1A^497^, but without ATP, either in kinase buffer (pH 7.4) or MES buffer (pH 6.5) containing 0.1 M NaCl, 5 mM MgCl_2_. Appropriate amounts of GST were added into each tube to compensate for the different kinase amounts. After chilling on ice, the reaction mixtures were ultracentrifuged as described in Fig. 1. The supernatants were blotted by using anti-α- or β-adaptin antibody. (*n = 2*). *(* ***C*** *) Release of multiple CCV proteins by Dyrk1A without phosphorylation.* The same samples from (*B*) at pH 7.4 were analyzed by using various antibodies as indicated. *Lanes* (−) and *1–5* represent the kinase concentrations at 0, 0.1, 0.25, 0.5, 1.0, and 2 µg/assay. One µg Dyrk1A/assay is equal to 4.2×10^−7^ M by assuming the enzyme purity as 100%.

Next, we studied the effect of Dyrk1A on dissociating the clathrin adaptor proteins from CCVs (Fig. 5*A*). AP180 was slightly released from CCVs in the absence of Dyrk1A (*lanes a, b*). Addition of both ATP and Dyrk1A clearly enhanced the dissociation of AP180 and β-adaptin (*lanes c, d*). Apparently, the mere presence of Dyrk1A also enhanced the AP180 release (*lane c*). Therefore, the Dyrk1A effect without enzymic action was further studied by using various concentrations of Dyrk1A in the absence of ATP (Fig. 5*B*). The basal release levels of α- and β-adaptins were higher in HEPES buffer at pH 7.4 (phosphorylation buffer) than in MES at pH 6.5 (CCV isolation buffer), and Dyrk1A was more effective in promoting the release of α- and β-adaptins at pH 7.4. Dyrk1A also dissociated AP180 and μ2-adaptin from CCVs in a kinase concentration–dependent manner (Fig. 5*C*). Thus, Dyrk1A promoted the release of clathrin-adaptor proteins by phosphorylation and physical interaction. However, Dyrk1A did not have any significant effect on dissociating CHC (Fig. 5*A,C*).

### Effect of Dyrk1A-Mediated Phosphorylation on Dissociation of Clathrin Adaptor Proteins

**Figure 6:**
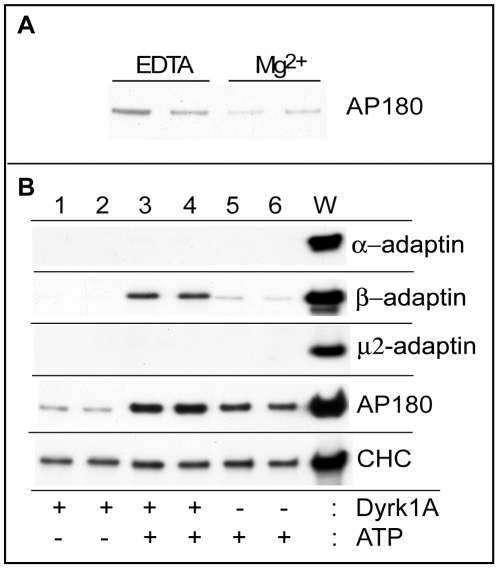
Effect of phosphorylation on dissociation of adaptor proteins in the presence of Mg^2+^. *(* ***A*** *) Effect of Mg^2+^ on AP180 dissociation*. CCVs (3 µg) were incubated with Dyrk1A and cold ATP as described in Fig. 5*A*, but with modified conditions including 75 mM HEPES (pH 7.0), 1 mg/ml BSA, and a mixture of phosphatase-inhibitors. After the reaction, the tubes were mixed with either 10 mM EDTA (*EDTA*) or with H_2_O (*Mg^2+^*) and ultracentrifuged. The resultant supernatant fractions were blotted with anti-AP180 antibody. (*n = 3*). Duplicated samples were shown. *(* ***B*** *) Effect of phosphorylation on adaptor protein dissociation.* CCVs were incubated as in *(A)* with ± Dyrk1A and ± ATP as indicated in the panel. The reaction mixtures were ultracentrifuged without adding EDTA. The same volumes of the supernatants and whole reaction mixtures (*W*) were immunoblotted using the indicated antibodies. Duplicated samples were shown. (*n = 1, various preliminary performances carried out to lead the final assay conditions are not included*).

Successive studies were carried out to estimate the effect of Dyrk1A-mediated phosphorylation on releasing the CCV-associated proteins. Because clathrin and its adaptor proteins dissociated spontaneously from CCVs (basal dissociation) under the standard phosphorylation conditions employed in this study, we sought assay conditions that could support Dyrk1A activity and provide a lower basal dissociation of the CCV-associated proteins. HEPES buffer at pH 7.0 appeared to meet both criteria; although MES buffer provided a more stable association of the adaptor proteins (Fig. 5*B*), MES buffer was avoided since it strongly inhibited Dyrk1A activity over a wide pH range. The basal dissociation of clathrin adaptor proteins could be further reduced if free Mg^2+^ was present in the mixtures throughout the assay process (Fig. 6*A*). Therefore, the following studies were performed with HEPES at pH 7.0 without adding EDTA at the end of the phosphorylation reaction. As seen in Fig. 6*B* (*lanes 1–6 & W*), both α- and μ-adaptins of the AP2 complex were not detected in the soluble fraction under any assay conditions when Mg^2+^ was present. On the other hand, the release of β-adaptin was markedly enhanced when CCVs were incubated with both Dyrk1A and ATP (*lanes 3 & 4*). The selective release of β-adaptin subunit indicates that Dyrk1A phosphorylation caused dissociation of β-adaptin from the α- and μ-subunits of the AP2 complex. The kinase itself did not appear to dissociate any of the adaptin subunits (*lanes 1 & 2*), while ATP alone released minimal amounts of β-adaptin (*lanes 5 & 6*).

Dissociation of AP180 from CCVs was also enhanced after incubation with Dyrk1A and ATP (Fig. 6*B*, *lanes 3 & 4*). However, unlike β-adaptin, either Dyrk1A or ATP alone also induced AP180 dissociation at some levels. It appears, therefore, that three factors affect the dissociation of AP180: ATP, Dyrk1A, and phosphorylation. By comparison, release of CHC was, again, unaffected under the experimental conditions employed.

### Phosphorylation of the Membrane-Unbound Form of AP180 and α- and β-Adaptins

**Figure 7:**
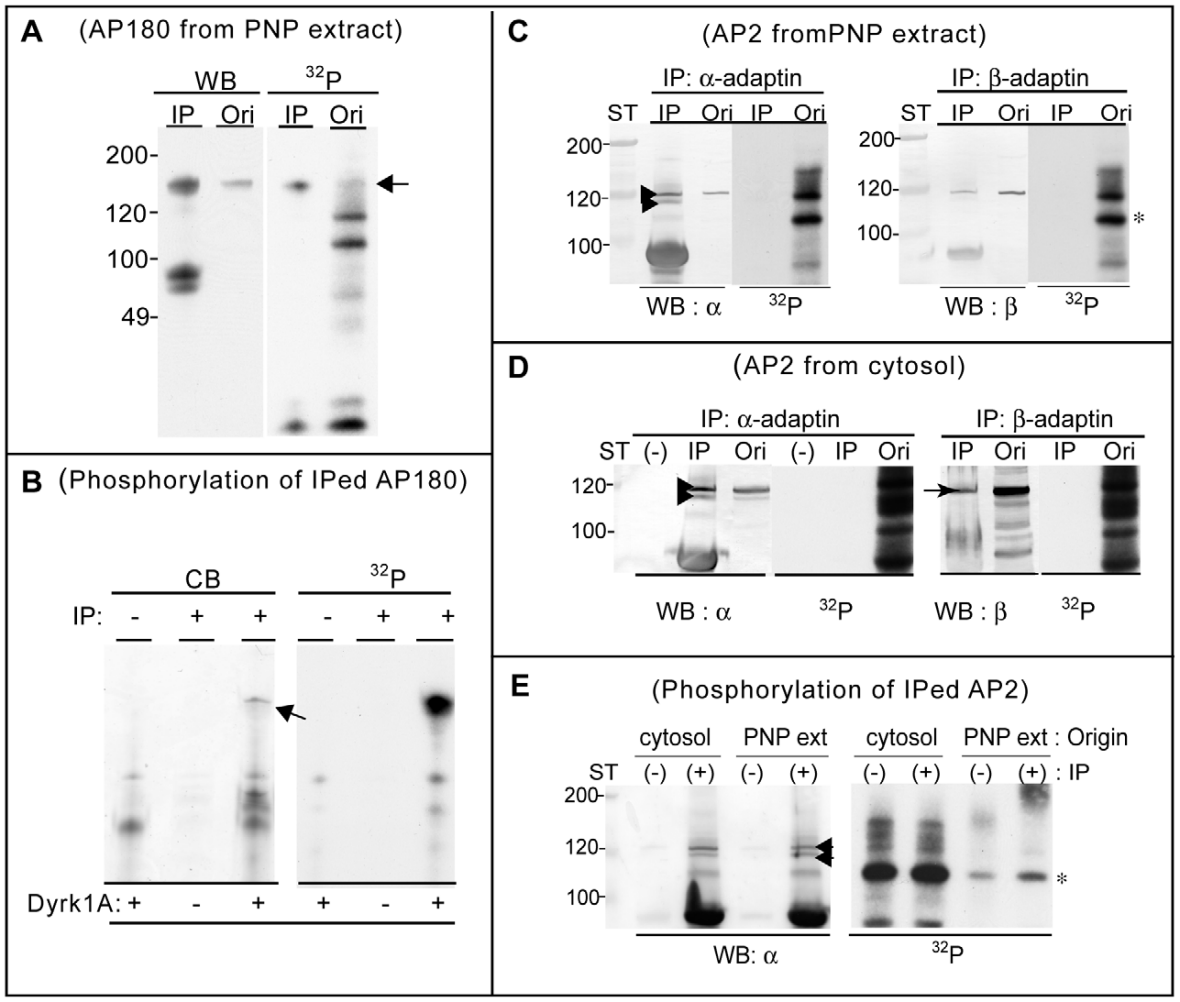
Phosphorylation of the membrane-unbound adaptor proteins. *(* ***A*** *) Immunoprecipitation of AP180 from the phosphorylated PNP extract*. The Tris-HCl extract from PNP (diluted, 300 µl) prepared as in *MATERIALS AND METHODS* was phosphorylated for 1 hr in a mixture containing 0.2 mM [γ-^32^P]-ATP, 9 µg Dyrk1A, 5 mM MgCl_2_, 0.12 M NaCl, 0.1 mM EGTA, and 0.2 mM DTT. After the reaction, the mixture was subjected to immunoprecipitation by using anti-AP180 antibody as in Fig. 3*B*. (*n = 2*). The immunoprecipitate (*IP*) and an aliquot of the original phosphorylation mixture (*Ori*) were subjected to immunoblotting (*WB*) and autoradiography (*^32^P*). (***B***) *Phosphorylation of the immunoprecipitated AP180*. The PNP extract was first subjected to immunoprecipitation with (+) and without (−) anti-AP180 antibody. The pellets of Protein A/G resins were washed three times with PBS-T and once with kinase buffer, and suspended in a small volume of kinase buffer (20 µl). Phosphorylation reaction was carried out in the presence of 0.2 mM [γ-^32^P]-ATP and Dyrk1A (2 µg) in a final liquid volume of 25 µl. Aliquots of the reaction mixtures were subjected to SDS-PAGE followed by Coomassie-Blue staining (*CB*) and autoradiography (*^32^P*). *Arrow*, AP180. (*n = 1*). (***C, D***) *Immunoprecipitation of α- and β-adaptins after phosphorylation reaction*. The PNP extract (***C***) and cytosol (***D***) were incubated with Dyrk1A and [^32^P]-ATP and subjected to immunoprecipitation with (*IP*) and without (−) corresponding antibodies. The immunoprecipitates and the aliquots of original reaction mixtures (*Ori*) were subjected to immunoblotting and autoradiography. (*n = 3*). (***E***) *Incubation of the immunoprecipitated adaptins with Dyrk1A*. Cytosol and the PNP extract were first subjected to immunoprecipitation without (−) and with (+) anti-α-adaptin antibody. The resultant precipitates were incubated with Dyrk1A and [γ-^32^P]-ATP followed by immunoblotting (*WB*) and autoradiography (*^32^P*) as described in (*B*). (*n = 1, various preliminary performances carried out to lead the final assay conditions are not included*). α, α-adaptin; β, β-adaptin; PNP-Ext, PNP extract; arrowheads, α-adaptin; *arrow*, β-adaptin; *, autophosphorylated Dyrk1A.

To further study the Dyrk1A-mediated regulation of the clathrin adaptor protein functions, we then tested if the adaptor proteins in their soluble forms were also phosphorylated by Dyrk1A. Two sources for the free form of the adaptor proteins from rat brains were employed for the experiments; a) cytosol and b) 0.5 M Tris-HCl extract from the post-nuclear precipitated fraction (*PNP extract*) diluted to the final Tris-HCl concentration to 0.125 M. Both AP180 and AP2 complexes were found in cytosol and in the PNP extract at comparable levels (not shown). However, the PNP extracts contained much lower concentration of total protein and many fewer protein bands phosphorylated by Dyrk1A (Fig. 7*A*). AP180 was immunoprecipitated equally well from cytosol and the PNP extract, giving the PNP extract an advantage for use as a starting material. Therefore, AP180 in the PNP extract was analyzed by the following two approaches; 1) immunoprecipitation of the adaptor proteins after phosphorylation reaction and 2) phosphorylation of the immunoprecipitated proteins. After incubating the extract with Dyrk1A and [^32^P]-ATP, the AP180 was clearly [^32^P]-labeled in the immunoprecipitates (Fig. 7*A*). Similarly, the AP180 immunoprecipitated from the PNP extract was [^32^P]-phosphorylated on Protein A/G resins by Dyrk1A (Fig. 7*B*). These results demonstrate that Dyrk1A phosphorylates AP180 both in the CCV bound- and unbound forms.

Consistent with the observation for the CCV extract (Fig. 3*F*), we found that the immunoprecipitation efficiencies of the AP2 complexes were rather poor from the PNP extracts. The immunoprecipitated α- and β-adaptins from the phosphorylated PNP extract did not contain ^32^P-phosphate (Fig. 7*C*). The radioactive band that migrated at a close proximity to the α- and β-adaptin bands in the original mixture did not match with these adaptins. The same anti-α and anti-β adaptin antibodies used in Fig. 3 failed to immunoprecipitate the AP2 complex from cytosol. Premixing the cytosol with 0.125 M Tris-HCl helped the immunoprecipitation to some extent. However, the adaptin subunits immunoprecipitated from the phosphorylated cytosol were not ^32^P-labeled (Fig. 7*D*). We next tested whether Dyrk1A could phosphorylate the immunoprecipitated AP2 complexes. After incubation of the immunoprecipitates with Dyrk1A and ^32^P-ATP, Dyrk1A (*) was the major band labeled with [^32^P]-phosphate. In contrast to the kinase bands, there was no ^32^P-labeled bands specific to the immunoprecipitation. From these results, we concluded that Dyrk1A phosphorylated only the membrane-bound form of α- and β-adaptins. Interestingly, the Protein A/G resins pre-incubated with cytosol enhanced [^32^P]-labeling of the Dyrk1A band, to the level much higher than those with the PNP extract, and the enhancement was independent to the presence of antibody (Fig. 7*E*). The observed enhancement could be due to autophosphorylation of the kinase stimulated by a co-factor(s) or mediated by other kinase(s), derived from cytosol and non-specifically bound to Protein A/G resins.

### Relative Ratios of Adaptin Subunits in the Soluble and Insoluble Fractions from Cultured Cells

**Figure 8:**
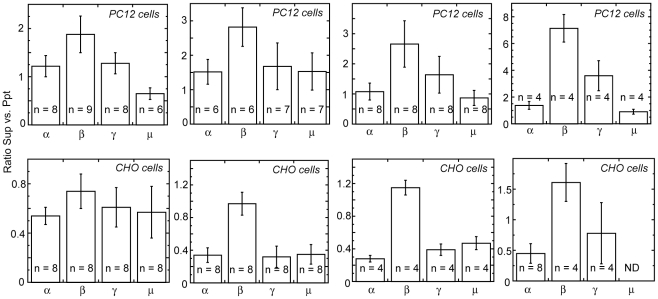
Ratios of adaptin subunits recovered in cytosol to the precipitated fractions from cultured cells. Cytosol (*Sup*) and precipitated (*Ppt*) fractions from CHO and PC12 cells were prepared as described in *MATERIALS AND METHODS*. SDS-PAGE was carried out by applying equal volumes of the soluble and insoluble fractions in each lane side-by-side. Immunoblotting was performed by using corresponding antibodies derived from mouse and rabbit. The first blots with mouse and rabbit antibodies were stripped (stripping buffer, PIERCE) and re-blotted with rabbit and mouse antibodies, respectively, for detecting other adaptin subunits in the same membranes. The antibodies used were mouse monoclonal antibodies against anti-α and γ-adaptins, rabbit monoclonal anti-β-adaptin antibody, and rabbit polyclonal anti-μ adaptin antibody. Each adaptin band in the *Sup* and *Ppt* was scanned, and the *Sup* to *Ppt* ratio was calculated. Four independent samples per each cell type were shown.

Our *in vitro* studies show that the Dyrk1A-mediated phosphorylation dissociates the adaptin subunits, and free β-adaptin can be released into cytoplasm *in vivo*. We expected that more β-adaptin could be recovered in cytosol than other adaptin subunits from the cells active in endocytosis. Therefore, the relative ratios of each adaptin subunit (α, β, γ, and μ) in cytosol (*Sup*) and the membrane bound (*Ppt*) fractions were estimated for multiple samples from CHO and PC12 cells. After scanning the immunoblots, the ratios of each adaptin subunit recovered in cytosol to precipitate were calculated. As summarized in Fig. 8, the ratios of β-adaptin in cytosol to precipitate were the highest among the adaptin subunits tested. In addition, these ratios of β-adaptin were clearly higher than those of γ-adaptin, thus excluding a possibility that the released β-adaptin was derived solely from the AP1 complex.

### Dyrk1A in the CCV Fraction

**Figure 9:**
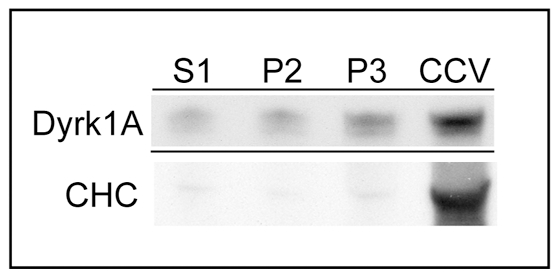
Relative kinase concentrations in the CCV fraction. Rat brain fractions S1, P2, P3 were prepared as described in *MATERIALS AND METHODS*. These fractions together with CCVs were subjected to SDS-PAGE followed by immunoblotting using 8D9 (*Dyrk1A*). Fifteen µg of protein were applied per each lane. After stripping, the same membrane was re-blotted with anti-CHC antibody. (*n = 1*, *various preliminary performances carried out to lead the final assay conditions are not included*).

We have demonstrated previously that Dyrk1A is able to bind CHC and co-assembled into clathrin cages under *in vitro* assays [20], which suggests the presence of this kinase in CCVs. Therefore, we examined the Dyrk1A level in the CCV preparation together with other subcellular fractions. Immunoblotting of S1 (sup at 1,000× g), P2 (ppt at 19,000× g), P3 (ppt at 100,000× g), and CCVs with anti-Dyrk1A antibody (8D9) revealed that the relative kinase levels gradually increased from S1, P2, P3 to CCVs, such that CCVs contained the highest level of Dyrk1A per unit protein (Fig. 9). Scanning of the immunoblot gave the ratios of Dyrk1A in P2, P3, and CCV as 1.3, 2.3, and 5.9, respectively, by normalizing S1 as 1. Re-blotting the same PVDF membrane with anti-CHC revealed that clathrin was strikingly accumulated in CCVs. By following the same approach as described above, the relative levels of CHC in P2, P3, and CCV were estimated to be 0.8, 2.1, and at least 52.2, respectively. Our results suggest that the Dyrk1A level is elevated in CCVs, but not particularly enriched there in proportion to CHC.

### Co-Localization of Clathrin and Dyrk1A in Brain Sections and Primary Cultured Neurons

**Figure 10:**
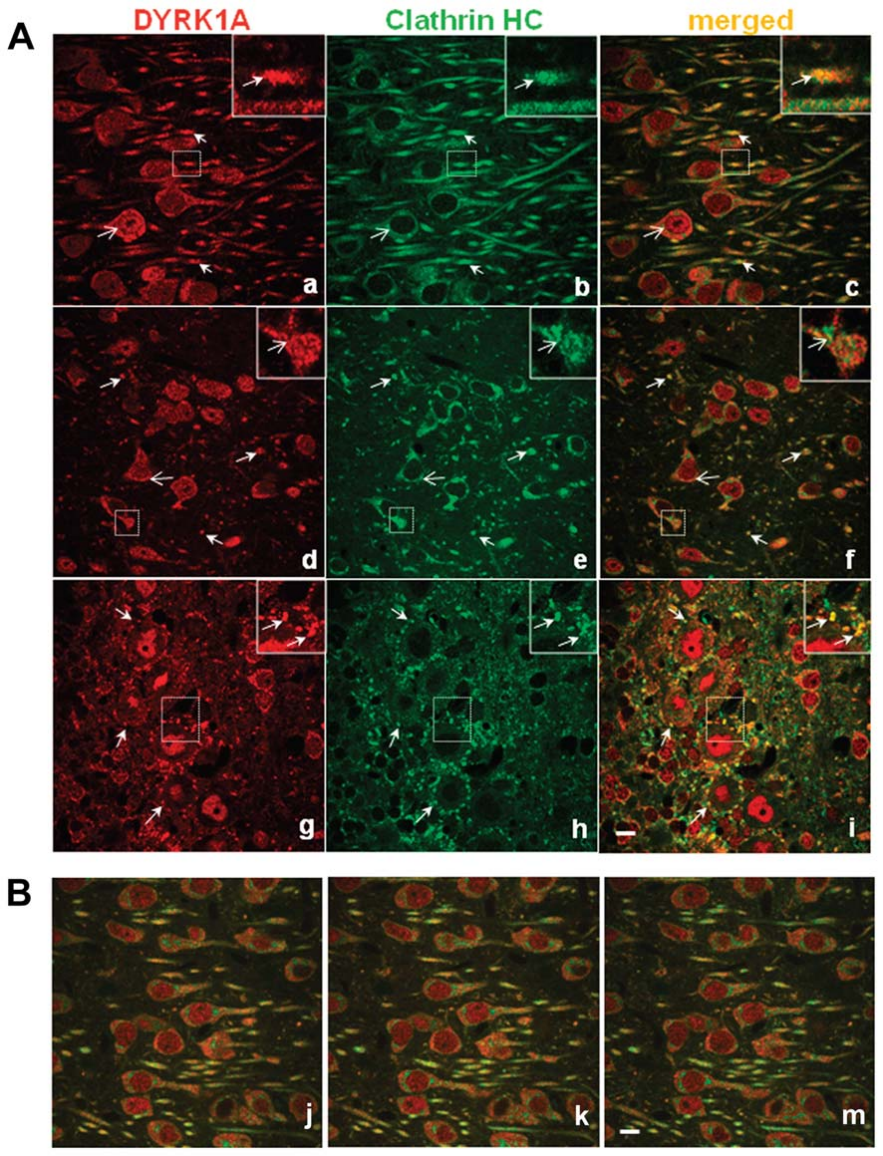
Co-localization of endogenous Dyrk1A and clathrin in the adult mouse brains. Forty micrometer sections of the mouse brain tissues were incubated with polyclonal anti-Dyrk1A (red, *a*, *d*, and *g*) and monoclonal anti-CHC (green, *b*, *e*, and *h*) antibodies as described in *MATERIALS AND METHODS*. Presented are Z-stack images collected from the subiculum (*a–c*), the medio-posterior thalamic nucleus (*d–f*), and individual confocal images from the cerebellar Purkinje cell layer (*g–i*). Structures showing co-localization of Dyrk1A and CHC are visible on merged images (yellow in *c, f, i*). The dotted squares shown in *a–i* are enlarged in the upper right corners. Scale bar = 10 µm. Panels (*j–m*) show three consecutive scanning images from subiculum. Pearson's correlation coefficients for three consecutive scanning images from subiculum and the medio-posterior thalamic nucleus were calculated as 0.439 and 0.499, respectively, by using NIH Image J with the JACoP plug-in [65].

We examined whether Dyrk1A is associated with CCVs *in vivo* by performing double immunostaining of various brain sections with anti-Dyrk1A and anti-CHC antibodies. Images captured by laser-scanning confocal microscopy are shown for the sections from the hippocampus (*a–c*), thalamus (*d–f*), and cerebellum (*g–i*) in Figure 10. Consistent with the observation that Dyrk1A has a nuclear localization sequence [2], some Dyrk1A was observed in the nucleus. This kinase was also found in soma (*open arrows*) and dendrites (*arrows*), which is consistent with the earlier results using mouse monoclonal antibodies 7F3 [10] and 7D10 [26]. Clathrin was detected in the perinuclear area of the cell soma (*open arrow*) and in dendrites and axons (arrows in *panels b,e*), but not in the nuclei. Both anti-Dyrk1A and clathrin antibodies visualized the punctated and vesicular-like structures. Co-localization of Dyrk1A with CHC in the soma of neurons was sparse (open arrows; *a–f* and the insets in *d–f*). In contrast, these proteins often co-localized in dendritic processes (arrows in *a–f* and the insets in *a–c*) in all brain regions examined. There were also structures containing either only Dyrk1A or clathrin within a single process (*c, f*). By analysing sequential confocal images collected at 0.15 µm steps along the Z-axis (*panels j–m*), the Dyrk1A and/or CHC-positive structures were found in various areas of the cytoplasm of dendrites, including sub-plasmalemmal regions. Dyrk1A was also found in some axonal terminals (*arrows* in *g*). The numerous axon terminals of basket cells forming contacts with Purkinje cell somata in the cerebellar cortex showed strong co-localization of Dyrk1A with clathrin (arrows in *g–i* and their insets).

**Figure 11:**
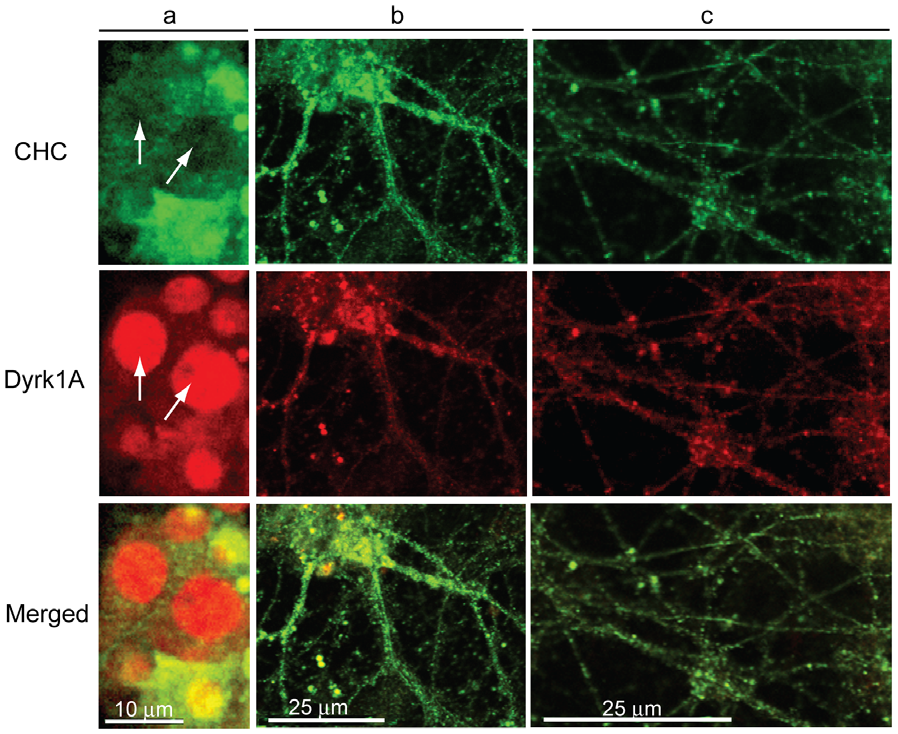
Localization of Dyrk1A and clathrin in the primary cultured neurons. E-18 rat hippocampal neurons were differentiated, fixed with 2% formaldehyde, and immunostained with monoclonal anti-CHC followed by Alexa Fluor 488 conjugated goat anti mouse IgG. After completion of CHC staining, the cells were incubated with monoclonal anti-Dyrk1A antibody (7F3), directly conjugated with Alexa Fluor 568 as described in *MATERIALS AND METHODS*. (*a*), single confocal images; (*b* and *c*), Z-stack images. (*n = 2*). Pearson's correlation coefficient for single scanning images was 0.645 as calculated as in Fig. 10.

Association of Dyrk1A with CCVs was also examined in primary cultured neurons from the E-18 rat hippocampus (Fig. 11). Again, both Dyrk1A and CHC were found as punctated structures in the soma (*a, b*) and in the processes (*b, c*) of the cultured cells. Co-localization of these two proteins was very prominent in the processes but was less significant in the soma of E-18 neurons. Immunostaining showed that Dyrk1A in the primary cultured neurons was more abundant in nuclei than in the cytoplasm. Individual confocal images collected along the Z-axis confirmed that clathrin was mostly absent in the nuclei (*arrows* in Fig. 11*a*). Thus, co-localization of Dyrk1A with CHC was evident in both mouse brain as well as cultured primary neurons from rat hippocampus. From the results in Figs. 9, 10, and 11 we conclude that *in vivo*, certain fractions of Dyrk1A are associated with CCVs either directly or indirectly.

## Discussion

We have identified five Dyrk1A substrate proteins in rat brain CCVs, MAP1A, MAP2, AP180, and α- and β-adaptins, and found that phosphorylation enhanced the release of these substrates from CCVs. Although several kinases are known to be associated with CCVs, Dyrk1A is the first kinase shown to promote the dissociation of adaptor proteins from CCVs.

### I. Phosphorylation of MAP1A and MAP2

#### a) Identification as Dyrk1A Substrates

The CCV preparations employed in the current study contained both MAP1A and MAP2, and our results indicate that both proteins are Dyrk1A substrates (Fig. 2). Based on consensus sequences for Dyrk1A phosphorylation, RPX(S/T)P and RX(S/T)P [1], rat MAP1A contains three potential Dyrk1A sites (RRT ^2059^P, RPAS ^2221^P, and RPS ^2546^P) in the heavy-chain. This is similar to rat MAP1B which contains multiple consensus Dyrk1A sites and phosphorylated by the kinase [29]. MAP2, on the other hand, does not contain any of the consensus sequences. However, the phosphorylation efficiencies of MAP1A and MAP2 in MTs were very similar (Fig. 2*C*). We believe that Dyrk1A can phosphorylate these MAPs equally in the CCV preparations as well. Our CCV preparations were relatively crude and contained tubulin; thus, MTs might exist as contaminants. Alternatively, because MAP1A has been identified as one of the proteins bound (directly or indirectly) on the α-appendage of the AP2 complex [30] and because the epsin N-terminal homology/AP180 N-terminal homology (ENTH/ANTH) domain has been shown to bind tubulin [31], the presence of MTs in the CCV preparations might be physiologically relevant.

#### b) Effect of Phosphorylation

We found that Dyrk1A phosphorylated the bound (or insoluble) form of MAPs in the CCV preparations and released the proteins into the solution (Fig. 4). Because MAPs are the proteins which promote MT assembly and stabilize MT dynamics [32], [33], [34], [35], [36], the Dyrk1A-mediated phosphorylation could have a destabilizing effect on MTs via dissociating MAP1A and MAP2. However, under similar assay conditions, the release of MAPs from the purified MT preparations was independent of Dyrk1A (data, not shown). Therefore, we speculate that the phosphorylation by Dyrk1A may disrupt the bindings between MAPs and the CCV-associated proteins, rather than the destabilizing MTs. In either case, CCVs could be released from the MT cytoskeleton after MAP phosphorylation. A recent report indicates that Dyrk1A is a priming kinase required for subsequent phosphorylation of MAP1B by GSK3β, which reduces MT stability [29]. We speculate that Dyrk1A could function as the priming kinase also for MAP1A and MAP2 as well and subsequently affects the MT dynamics via GSK3β action.

### II. Phosphorylation of Clathrin-Adaptor Proteins by Dyrk1A

#### a) Identification of Proteins

Similar to MAP2, AP180 does not contain any sequences matching the consensus Dyrk1A phosphorylation site, but immunoprecipitation analysis confirmed AP180 as a Dyrk1A substrate associated with brain CCVs (Fig. 3). We conclude that Dyrk1A can phosphorylate AP180 both in membrane-bound and unbound forms, because *1)* AP180 dissociation from CCVs was clearly less if phosphorylation did not take a place (Fig. 6*B*), *2)* significant amounts of the [^32^P]-labeled AP180 were still associated with the CCVs (Fig. 1), and *3)* Dyrk1A phosphorylated AP180 in cytosol (not shown) and in the PNP extracts (Fig. 7).

Dyrk1A phosphorylated both α- and β-adaptins almost exclusively at serine residue(s) (not shown). Rat α1- and α2-adaptin isoforms of the AP2 complexes contain one (at S^518^) and two (at S^179^ and S^518^) potential kinase sites, respectively, whereas the β1- and β2-adaptin isoforms do not contain any consensus kinase site at serine residue. Because the anti-β-adaptin antibody used in the current study recognizes both β1 and β2 isoforms of the AP1 and AP2 complexes, respectively, it is not yet possible to conclude whether Dyrk1A phosphorylated β1, β2, or both isoforms. Judging from their extensively homologous amino acid sequences, both β-adaptin isoforms could be the Dyrk1A substrates. In contrast to AP180, α- and β-adaptins were phosphorylated by the kinase only when they were associated with CCVs. We assume that the Dyrk1A sites of α- and β-adaptins in the soluble form of AP2 could be blocked sterically through interacting with its binding protein(s). Alternatively, the kinase sites are embedded inside the AP2 complex; the conformational changes caused by binding to the membrane-lipids [37] could expose the sites. In addition to the complete failure in their phosphorylation, we also found it difficult to immunoprecipitate the AP2 complexes from cytosol. Although we employed multiple commercial monoclonal and polyclonal antibodies against α- and β-adaptins (immunogens were; peptide *aa*38–215 and purified AP2 complex for α-adaptin; peptides *aa*650–949 and *aa*75–245 as well as purified AP2 complex for β-adaptin), all failed. Addition of Tris-HCl (at 0.125 M) helped somewhat to pull down the complex from the cytosol (Fig. 7*D,E*). Tris-HCl at high concentrations might loosen the conformation (or interaction with binding proteins) of each adaptin subunit in the AP2 complexes. Nonetheless, the causes for the failure of immunoprecipitation and phosphorylation appear to be unrelated because the immunoprecipitated AP2 could not be phosphorylated by Dyrk1A (Fig. 7*E*).

#### b) Effect of Phosphorylation on the Clathrin Adaptor Proteins

To our surprise, the Dyrk1A-mediated phosphorylation caused separation of β-adaptin from the α-adaptin subunit (Fig. 3*C–F*), and only β-adaptin was released from the CCV membranes when free Mg^2+^ was present in the system (Fig. 6). These results suggest that phosphorylation of α- and β-adaptins by Dyrk1A induces disassembly of the AP2 complex on the CCV membranes and that when Mg^2+^ tightens the binding of α- and μ-adaptins to the membrane lipids (PIP_2_), β-adaptin becomes free from the membranes. Because the anti-β-adaptin antibody we employed reacts with both β1- and β2-adaptin isoforms and cannot distinguish between the AP1 and AP2 complexes, one might argue that the phosphorylation simply affected the membrane binding of AP1 complex and that what was seen solely reflected the release of AP1 complex from CCV membranes. However, we strongly believe that this was not the case because α-adaptin failed to co-precipitate together with β-adaptin only after phosphorylation with Dyrk1A (Fig. 3). In support of our argument from the *in vitro* studies, the ratios of β-adaptin recovered in cytosol to that in the precipitate from CHO and PC12 cells were indeed higher than any other adaptin subunits (Fig. 8). Variations in the α- to β-adaptin ratios were also seen in a report where brain cytosol was used for the binding to various phospholipids [38]. The physiological significance of disassembling the AP2 complex is not clear. The disassembly may moderate the pool size of the AP2 complexes available for the next endocytosis cycle, or may provide synaptojanin better access to the membrane PIP_2_ after β-adaptin is removed and, thus, accelerating the release of the PIP_2_-binding proteins from the vesicle membranes. We expect that the adaptin subunits released in the cytoplasm could re-assemble back into AP2 complexes after dephosphorylation by phosphatases such as calcineurin. Further studies are required to address these possibilities.

Similar to the AP2 complex, Dyrk1A phosphorylation of AP180 accelerated its release from the CCV membranes (Figs. 5 & 6). Yet the majority of the phosphorylated AP180 (as well as α- and β-adaptins) remained associated with the CCV membranes as seen in Fig. 1. These proteins may need to be phosphorylated at multiple sites to effect dissociation from the membranes. Alternatively, both AP180 and the AP2 complex must be phosphorylated simultaneously for their dissociation. AP180 binds to both α- and β-adaptins [39]. The fact that, in the presence of Mg^2+^, phosphorylation released AP180 and β-adaptin but not α-adaptin from CCVs (Fig. 6*B*) suggests that the binding between AP180 and the AP2 complex was disrupted by the Dyrk1A-mediated phosphorylation. A more complete understanding of the basis for the altered binding to the CCV membrane will require identification of the actual phosphorylation sites for these proteins.

### III. Phosphorylation of the Endocytic Proteins

#### a) The Kinases Associated with Isolated CCVs

Multiple protein kinases have been found to be associated with isolated CCVs: CKII, CVAK104, and auxilin2/GAK [23], [28], [40], [41], [42], [43], [44]. Apparently, μ2-adaptin is the only CCV-associated protein phosphorylated by endogenous kinase when intact CCVs are incubated with [γ-^32^P]-ATP (Fig. 1), consistent with a previous report [28]. This phosphorylation reaction is mediated by auxilin2/GAK that co-purifies with CCVs [28], [43]. Phosphorylation by other CCV-associated kinases seems to be less relevant; first, the CCV-bound CKII is inactivated by binding to phosphatidylinositols of the CCV membrane [45]. Secondly, although CVAK104 binds directly to both clathrin triskelia and the isolated AP2 complex and co-fractionates with the adaptor proteins, this kinase phosphorylates β2-adaptin only when isolated AP2 is employed [44].

We have shown here that Dyrk1A was also associated with brain CCVs (Fig. 9). In various neurons from mouse brain sections and rat primary cultures, certain fractions of the endogenous Dyrk1A were co-localized with clathrin (Figs. 10,11). Based upon our *in vitro* analysis, recombinant Dyrk1A could bind directly to CHC, endophilin 1, and dynamin [20], but apparently did not exert effects on the assembly of clathrin cages [20] and on the disassembly of clathrin from CCVs (Figs. 5,6). The exogenous Dyrk1A also seemed to form complexes with AP180 and AP2 on the CCV membrane because Dyrk1A alone (without ATP) could dissociate these adaptors from the membranes in a concentration-dependent manner when EDTA was added at the end of the incubation (Fig. 5*B,C*). Such complexes appeared to remain associated with CCVs in the presence of Mg^2+^ (Fig. 6). Because Dyrk1A was not accumulated in CCVs at the same ratio as CHC (Fig. 9) and because certain fractions of the CHC-positive structures did not contain Dyrk1A (Fig. 10), we speculate that the binding between Dyrk1A and CCVs *in vivo* is a transitory event and can be influenced by other factor(s). This could explain why only a fraction of the CCVs contained Dyrk1A. Alternatively, two distinct types of CCVs, one with and the other without Dyrk1A, may be formed at the discrete endocytosis sites (Fig. 10, *panel i*). Either way, the endogenous kinase alone was insufficient to phosphorylate the substrates in the isolated CCVs to significant levels (*Fig. 1*). We do not know the reason for such insufficiencies. As reported for CKII [45], the CCV-associated Dyrk1A must also be inactivated by some mechanism(s). Further studies will be required to address this point.

#### b) Regulation of CCV Formation

Phosphorylation may regulate the assembly of CCVs both positively and negatively. Phosphorylation of the μ2-adaptin is required for endocytosis by enhancing cargo recruitment into coated pits [46]. CHC is phosphorylated by *Src* kinase at Tyr^1477^ after EGF stimulation of A431 cells [47], and inhibition of the phosphorylation prevents redistribution of clathrin and delays EGF receptor endocytosis. In contrast, several proteins at nerve terminals (dynamin 1, amphiphysin 1 and 2, synaptojanin, AP180, epsin, and Eps15) are recognized as dephosphins, which are essential for synaptic vesicle endocytosis and are rapidly and coordinately dephosphorylated by calcineurin, Ca^2+^-calmodulin–dependent phosphatase, after Ca^2+^ influx [19]. Multiple kinases are reported for phosphorylating these dephosphins; PK C, CDKs [19], GSK 3 [48], and Dyrk1A [12], [13], [14]. In addition, CKII phosphorylates α- and β-adaptins and AP180. The phosphorylation of α- and β-adaptins occurs within their hinge regions and reduces their clathrin binding *in vitro*, whereas that of AP180 reduces its binding to AP2 complex, which in turn lowers the co-operative clathrin assembly activity of these adaptors [49]. Apparently CKII phosphorylates the unbound form of the adaptor proteins [49] and it is not clear whether the exogenous CKII in an excess amount can phosphorylate the CCV-associated substrates. In this regard, Dyrk1A is the first identified kinase that is capable of phosphorylating clathrin adaptors bound to the intact CCVs and to dissociate them from CCV membranes.

**Figure 12:**
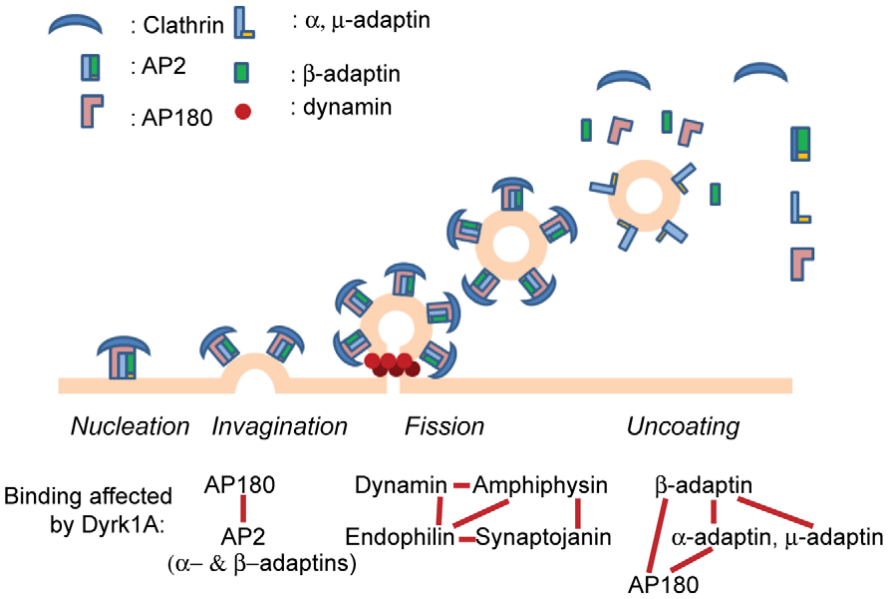
Proposed Dyrk1A functions in regulating synaptic vesicle endocytosis. Dyrk1A phosphorylated AP180 in cytosol. The phosphorylated AP180 may decrease its binding affinity to the AP2 complex; we hypothesize here that the decrease in such binding affinity would reduce recruitment of clathrin at the *nucleation* and *invagination* sites. Phosphorylation of dynamin 1, amphiphysin 1, and synaptojanin 1 at their PRD reduces the interaction between the PRDs and the SH3 domains of amphiphysin and endophilin. This likely slows down the *invagination* and *fission* steps of synaptic vesicle formation. Once endocytosed, the vesicle-associated proteins are quickly removed from the membranes (*uncoating*). The Dyrk1A-mediated phosphorylation releases first AP180 and β-adaptin from the vesicle membranes, while both α- and μ-adaptin subunits remain bound with the membranes. Additional factor(s) are required to release the membrane-bound subunits. Clathrin release from the vesicles is independent from Dyrk1A. We speculate that the adaptin subunits released in cytoplasm may reassemble into the AP2 complex after dephosphorylation.

We propose that Dyrk1A affects multiple steps in regulating synaptic vesicle endocytosis (Fig. 12). The fact that AP180, a brain-specific adaptor protein, was phosphorylated by Dyrk1A not only in the membrane-bound form but also the membrane-free form may imply that Dyrk1A affects not only dissociation of proteins from synaptic vesicles (*uncoating*) but also inhibits assembly of endocytic proteins at the vesicle formation sites (*nucleation/invagination*). We speculate that the phosphorylated AP180 in cytoplasm would slow a recruiting process of the AP2 complex and clathrin at their assembly sites. Phosphorylation of dynamin 1, amphiphysin 1, and synaptojanin 1 by Dyrk1A within their PRD modifies the PRD binding to the SH3 domain of amphiphysin and endophilin [12], [13], [14]. This is expected to reduce the invagination/fission stages of CCVs. From our current results, it is clear that Dyrk1A phosphorylation enhances the uncoating step of the endocytosed synaptic vesicles. The release of AP180 and β-adaptin might be mediated by reductions in the binding affinities between AP180 and AP2, between AP180 and membrane lipids, and among the adaptin subunits of the AP2 complex. Uncoating of clathrin-adaptor proteins has been proposed to be mediated by dephosphorylation of PIP_2_ via phosphatidylinositol 5′-phosphatase activity of synaptojanin [50], [51], [52]. However, the involvement of synaptojanin for the observed adaptor release is highly unlikely in our system because this protein was not detected in our CCV preparations and the Dyrk1A-mediated phosphorylation of synaptojanin 1 has no effect on its 5′-phosphatase activity [14]. Uncoating of clathrin, on the other hand, is mediated by auxilin and Hsc70 in an ATP-dependent manner [53], [54], [55]. Because clathrin dissociation may take place prior to dissociation of adaptor proteins, further studies are necessary to evaluate the effect of clathrin molecules on the Dyrk1A-mediated phosphorylation and dissociation of the adaptor proteins from CCVs. In the current studies, we employed the truncated form of Dyrk1A (Dyrk1A^497^) missing the C-terminal domain from aa498 to aa763 for *in vitro* assays. This was done because the truncated form (1) is highly purified in contrast to the full-length protein which always contains variously degraded kinase products [20], [56] and/or contaminants and (2) exhibits the similar kinase activity as the full-length protein [21]. Indeed, we confirmed that all kinase preparations (full length Dyrk1A, GST-full-length Dyrk1A, and GST-Dyrk1A^497^) phosphorylated the CCV-associated substrates very similarly (Figure S1). Although the role of the C-terminal domain in regulation of Dyrk1A functions is unclear, removal of the domain did not seem to affect the substrate specificity of Dyrk1A, at least when CCVs were used. It is noteworthy that because we have identified these substrates by *in vitro* assays using recombinant kinase, the findings need to be confirmed *in vivo* through other techniques, such as kinase-site specific antibodies.

Both Dyrk1A and the calcineurin inhibitor RCAN1/DSCR1/MCIP1 are encoded within the DS critical region [57], [58], [59], and their expression levels are elevated in individuals with DS [60], [61], [62], [63]. Dyrk1A is expressed strongly in the central nervous system of vertebrates, and many endocytic proteins expressed in brain are substrates for Dyrk1A. Indeed, over-expression of Dyrk1A seems to impair synaptic vesicle endocytosis [64]. Aberrant phosphorylation of multiple endocytic proteins would alter the cycling of synaptic vesicle endocytosis-neurotransmitter release, which in turn may alter plasticity of neurons and may result in mental retardation observed in Down syndrome.

## Supporting Information

Figure S1 **Phosphorylation of the CCV-Associated Proteins by Full-Length and Truncated Dyrk1A.** Four different CCV preparations (a–d) were incubated with full-length Dyrk1A (Dyrk^FL^), GST-full-length Dyrk1A (GST-Dyrk^FL^), or GST-truncated Dyrk1A (GST-Dyrk^497^) under the conditions as described in Fig. 1. The ultracentrifugation step was omitted, and the reaction mixtures were directly subjected to SDS-PAGE followed by autoradiography. Two distinct kinase preparations (#1 and #2) were used for each Dyrk^FL^ and GST-Dyrk^FL^. (***A***) CCV (preparation a, 12 µg/assay) was incubated with 1.25 µg each of Dyrk^FL^ (#1) and GST-Dyrk^497^. The reaction mixtures were subjected to SDS-PAGE using 7% acrylamide-0.128% *bis*-acrylamide gels as in Fig. 1. In this panel, the two lanes with Dyrk^FL^ were exposed twice as long as those with GST-Dyrk^497^. Under our SDS-PAGE conditions, Dyrk^FL^ migrated with an apparent molecular weight (∼115 kDa) greater than the calculated value of Dyrk^FL^ (87 kDa) for both endogenous (expressed in rat brain) and recombinant kinases. (***B***) Two CCV preparations (b and c; 7 and 14 µg, respectively) were phosphorylated by 0.5 µg each of GST-Dyrk^FL^ (#1) and GST-Dyrk^497^. To avoid co-migration of the autophosphorylated Dyrk1A band and band 4, SDS-PAGE was carried out using 7% acrylamide-0.22% *bis*-acrylamide gels. Autoradiogram is shown. All lanes have the same exposure time. The phosphorylated bands 1+2, 3, and 4 were scanned, and the relative ratios of the ^32^P-labeled bands 1+2 to bands 3 and 4 were calculated for each CCV preparation (b & c) phosphorylated with either truncated or full-length Dyrk1A; the ratios were 1∶0.69∶0.75 and 1∶0.73∶0.78 for CCV-b with truncated and full-length Dyrk1A, respectively. Similarly, the ratios were 1∶0.70∶0.54 and 1∶0.80∶0.61 for CCV-c with truncated and full-length Dyrk1A, respectively. (***C***) CCVs (preparation d) were incubated with Dyrk^FL^ (#2), GST-Dyrk^FL^ (#2), or GST-Dyrk^497^, and subjected to SDS-PAGE as described in (***B***). In the panel, the CCV lanes with Dyrk^FL^ were exposed twice as long than those by GST-Dyrk^FL^ and GST-Dyrk^497^. The phosphorylation patterns of bands 1–4 were very similar with all three kinase preparations. Relative ratios of phosphorylated bands 1+2 to bands 3 and 4 were 1∶0.74∶0.53, 1∶0.84∶0.68, and 1∶0.71∶0.49 for phosphorylation with Dyrk^FL^, GST-Dyrk^FL^, and GST-Dyrk^497^, respectively. Thus, full-length and the truncated forms of Dyrk1A recognize the CCV-associated substrates with a similar manner. For the CCVs with GST-Dyrk^FL^, the ratios were not normalized for the autophosphorylated kinase band. Note that full-length kinases showed much higher autophosphorylation levels than that of GST-Dyrk^497^. This is because the C-terminal domain contains multiple autophosphorylation sites and truncated kinase should be less autophosphorylated than the full-length kinase. Reduction in the autophosphorylation levels when incubated with the substrates may indicate that the autophosphorylation of Dyrk1A is in intermolecular, not intramolecular, event. *, autophosphorylated GST-Dyrk^497^.
(DOC)
