# Supplementary figures and images for: Phosphorylation by Dyrk1A of Clathrin Coated Vesicle-Associated Proteins: Identification of the Substrate Proteins and the Effects of Phosphorylation
Source: PLoS One. 2012 Apr 13;7(4):e34845. doi: 10.1371/journal.pone.0034845 (PMC3325943; doi:10.1371/journal.pone.0034845)

**
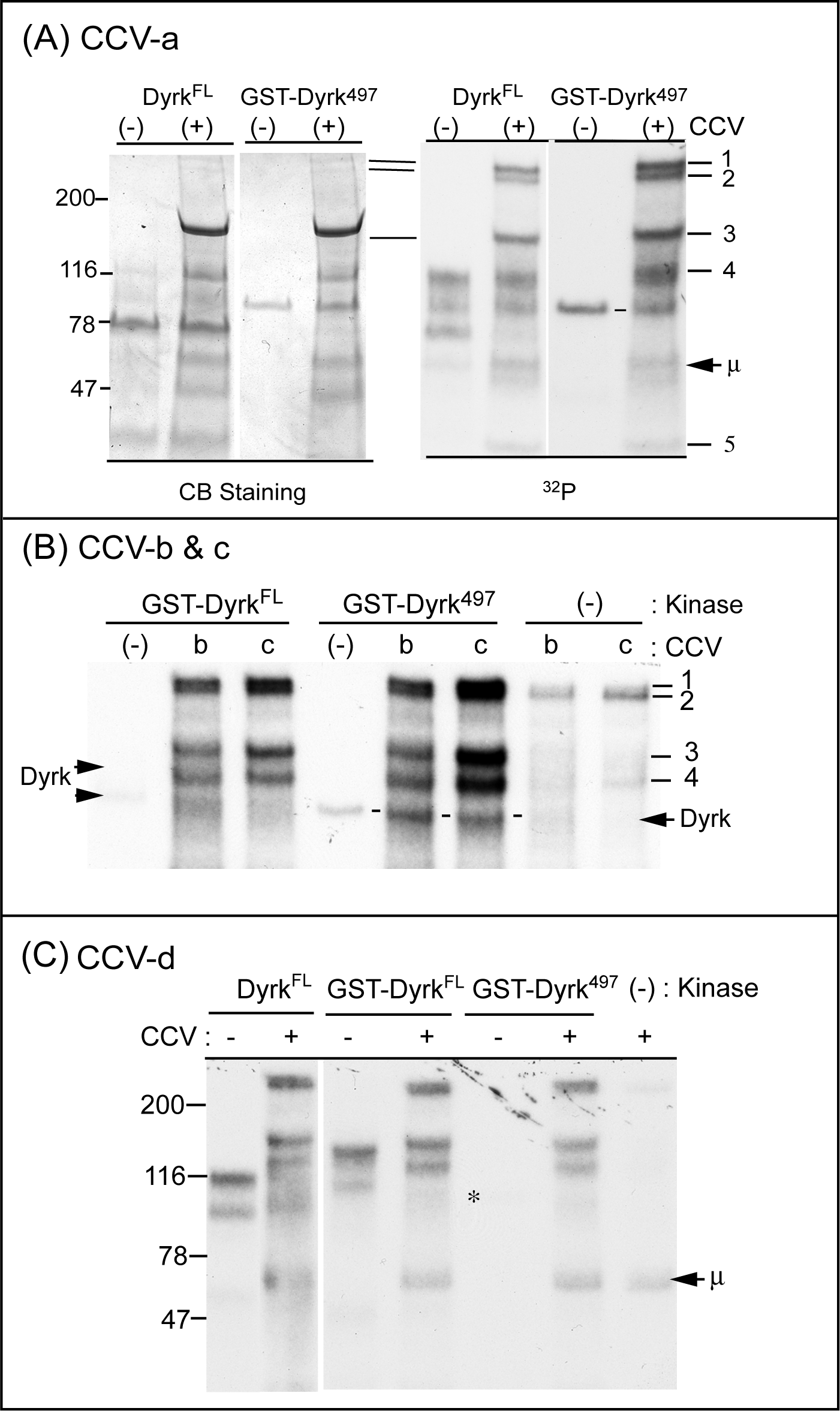
**

Supplement: Figure S1 — Phosphorylation of the CCV-Associated Proteins by Full-Length and Truncated Dyrk1A. Four different CCV preparations (a–d) were incubated with full-length Dyrk1A (DyrkFL), GST-full-length Dyrk1A (GST-DyrkFL), or GST-truncated Dyrk1A (GST-Dyrk497) under the conditions as described in Fig. 1. The ultracentrifugation step was omitted, and the reaction mixtures were directly subjected to SDS-PAGE followed by autoradiography. Two distinct kinase preparations (#1 and #2) were used for each DyrkFL and GST-DyrkFL. (A) CCV (preparation a, 12 µg/assay) was incubated with 1.25 µg each of DyrkFL (#1) and GST-Dyrk497. The reaction mixtures were subjected to SDS-PAGE using 7% acrylamide-0.128% bis-acrylamide gels as in Fig. 1. In this panel, the two lanes with DyrkFL were exposed twice as long as those with GST-Dyrk497. Under our SDS-PAGE conditions, DyrkFL migrated with an apparent molecular weight (∼115 kDa) greater than the calculated value of DyrkFL (87 kDa) for both endogenous (expressed in rat brain) and recombinant kinases. (B) Two CCV preparations (b and c; 7 and 14 µg, respectively) were phosphorylated by 0.5 µg each of GST-DyrkFL (#1) and GST-Dyrk497. To avoid co-migration of the autophosphorylated Dyrk1A band and band 4, SDS-PAGE was carried out using 7% acrylamide-0.22% bis-acrylamide gels. Autoradiogram is shown. All lanes have the same exposure time. The phosphorylated bands 1+2, 3, and 4 were scanned, and the relative ratios of the 32P-labeled bands 1+2 to bands 3 and 4 were calculated for each CCV preparation (b & c) phosphorylated with either truncated or full-length Dyrk1A; the ratios were 1∶0.69∶0.75 and 1∶0.73∶0.78 for CCV-b with truncated and full-length Dyrk1A, respectively. Similarly, the ratios were 1∶0.70∶0.54 and 1∶0.80∶0.61 for CCV-c with truncated and full-length Dyrk1A, respectively. (C) CCVs (preparation d) were incubated with DyrkFL (#2), GST-DyrkFL (#2), or GST-Dyrk497, and subjected to SDS-PAGE as described in (B). In the panel, the CCV lanes with [file pone.0034845.s001.doc]
